# Identification of genetic variants associated with idiopathic inflammatory myopathies via cross-trait analysis with B cell lymphomas

**DOI:** 10.1016/j.xhgg.2026.100658

**Published:** 2026-07-24

**Authors:** Weng Ian Che, James N. Jarvis, Anton Öberg Sysojev, Catherine Zhu, Karina Patasova, International Lymphoma Epidemiology Consortium (InterLymph), Sonja Berndt, Parveen Bhatti, Lauren Teras, Alain Monnereau, Roel Vermeulen, Delphine Casabonne, Alexandra Nieters, James McKay, Brenda Birmann, Pierluigi Cocco, James Cerhan, Brian Link, Roger Milne, Harindra Jayasekara, Vijai Joseph, Kenneth Offit, Nathaniel Rothman, Claire Vajdic, Alan Arslan, Mark Purdue, Henrik Hjalgrim, Paige Bracci, Eleanor Kane, Lesley Tinker, Qing Lan, Susan Slager, Immaculata De Vivo, Gilles Salles, Nicola Camp, IMACS Genetics Group (MYOGEN), Chris Amos, Olivier Benveniste, Hector Chinoy, Jan L. De Bleecker, Boel De Paepe, Andrea Doria, Zoltan Geiger, Peter Gregersen, Michael G. Hanna, Vidya Limaye, Pedro Machado, Pernille Mathiesen, Britta Maurer, Frederick W. Miller, Øyvind Molberg, Lauren Pachman, Leonid Padyukov, Timothy Radstake, Ann M. Reed, Lisa G. Rider, Simon Rothwell, Adam Schiffenbauer, Albert Selva-O’Callaghan, Jiri Vencovksy, Lucy R. Wedderburn, Karin E. Smedby, Ingrid E. Lundberg, Helga Westerlind, Janine A. Lamb, Marie Holmqvist

**Affiliations:** 1 Department of Public Health and Medicinal Administration, Faculty of Health Sciences, University of Macau, Macau, Macau SAR, China; 2 Department of Pediatrics and Center for Indigenous Health, University of Washington School of Medicine, Seattle, WA, USA; 3 Clinical Epidemiology Division, Department of Medicine, Solna, Karolinska Institutet, Stockholm, Sweden; 4 Institute for Clinical and Translational Research, Baylor College of Medicine, Houston, TX, USA; 5 Center for Molecular Medicine, Department of Medicine, Solna, Karolinska Institutet, Stockholm, Sweden; 6 Translational and Clinical Research Institute, Newcastle University, Newcastle upon Tyne, UK; 7 Population Health Sciences Institute, Newcastle University, Newcastle upon Tyne, UK; 8 Hematology Center, Karolinska University Hospital, Stockholm, Sweden; 9 Division of Rheumatology, Department of Medicine, Solna, Karolinska Institutet, Stockholm, Sweden; 10 ME Gastro, Derm and Rheuma, Theme Inflammation and Aging, Karolinska University Hospital, Stockholm, Sweden; 11 Epidemiology and Public Health Group, School of Health Sciences, The University of Manchester, Manchester, UK

**Keywords:** idiopathic inflammatory myopathy, dermatomyositis, polymyositis, genetics, single-nucleotide polymorphism, genome-wide association study, B cell lymphomas, conditional false discovery rate

## Abstract

The genetic architecture of idiopathic inflammatory myopathies (IIMs) remains incompletely defined. When increasing sample size is not feasible, cross-trait analysis of genetically correlated diseases offers an effective strategy for discovering risk loci. Using summary statistics of IIM and B cell lymphoma subtypes, we applied conditional false discovery rate (condFDR) and multi-trait analysis of genome-wide association studies (GWASs) (MTAG) to detect genetic associations with IIM risk. Single-nucleotide polymorphisms (SNPs) outside the human leukocyte antigen (HLA) region meeting significance thresholds (condFDR < 0.01 or *p* <5 × 10^−8^ for MTAG) were clumped and subjected to both functional annotation in FUMA and Gene Ontology (GO) biological process enrichment analysis using clusterProfiler. We identified six previously unreported loci, including three associated with dermatomyositis (chr12:58674304T>C, chr13:110799415C>T, and chr17:38103285G>A (hg19)) and three associated with polymyositis (PM) (chr4:971496T>C, chr6:396321C>T, and chr6:32650631C>A). All non-HLA loci act as expression quantitative trait loci (eQTL) or localize within enhancer regions. Notably, these include *cis*-eQTLs for known IIM risk-associated genes (*GSDMB*, *DGKQ*, *SLC26A1*, and *IDUA*) as well as genes implicated in synaptic vesicle cycle (SVC) pathways, immune regulation (*IKZF3*, *ORMDL3*, *IRF4*, *DUSP22*, and *SPON2*), protein homeostasis (*ATP23* and *PSMD3*), lipid metabolism (*PGAP3*, *ORMDL3*, *STARD3*, and *DGKQ*), and myopathy (*COL4A1*). GO analysis revealed significance for SVC pathways in PM (FDR < 0.05). These findings advance our understanding of IIM pathogenesis from a genetic perspective and highlight candidate regulatory variants for further mechanistic investigation.

## Teaser

Using genetic information from B cell lymphomas, we identified three and two unreported non-human leukocyte antigen loci for dermatomyositis and polymyositis, respectively. These loci show regulatory evidence in genes involved in synaptic vesicle cycling, immune-related activities, protein homeostasis, lipid metabolism, and myopathy, advancing our understanding of disease genetics.

## Introduction

Idiopathic inflammatory myopathies (IIMs) encompass a spectrum of rare rheumatic diseases characterized by diverse phenotypes, with most cases sharing muscle inflammation to varying extent as a common feature. Dermatomyositis (DM) and polymyositis (PM) are the two broad clinical subtypes traditionally defined based on the Bohan and Peter criteria, with PM being distinct from DM by the absence of skin manifestations. In recent years, the discovery of IIM-related autoantibodies linked to distinct clinical features and outcomes has led to the recognition of additional clinical subtypes. Many PM cases defined in previous studies could actually represent other IIM subtypes, including anti-synthetase syndrome (ASyS), immune-mediated necrotizing myopathy (IMNM), inclusion body myositis (IBM), and overlap myositis.^1^

While the pathogenesis of IIMs remains unclear, it has been suggested that IIMs may be triggered by environmental exposures in genetically susceptible individuals.^1^ Genome-wide association studies (GWASs) and fine-mapping studies performed with ImmunoChip in patients with IIMs published in the last decade have opened a door to understanding the genetic contribution to disease development, identifying alleles in the human leukocyte antigen (HLA) 8.1 ancestral haplotype as the strongest genetic risk factors.^2^^,^^3^ However, a gap in heritability exists, evidenced by the difference between the family-based heritability (22%) and ImmunoChip single-nucleotide polymorphism (SNP)-based heritability (5.5% for DM and 8.3% for PM).^4^^,^^5^ This discrepancy suggests that additional genetic variants associated with IIM risk remain unidentified. Discovering mild to moderate genetic associations with IIM risk may require larger sample sizes than previous genetic studies, which is challenging for such a rare disease. Nevertheless, researchers have successfully identified numerous non-HLA genetic variants linked to the risk of IIMs through meta-analysis of already genotyped SNPs from related rheumatic diseases, genome-wide imputation, and cross-trait analysis of GWAS summary statistics with immune-related diseases.^6^^,^^7^^,^^8^^,^^9^

B cell lymphomas, malignancies arising from different stages of B lymphocyte maturation, exhibit shared immunogenetic characteristics with IIMs and have been consistently linked to DM and PM.^1^^,^^10^^,^^11^^,^^12^ While significant associations with diffuse large B cell lymphoma (DLBCL) and follicular lymphoma (FL) have been reported in patients with IIMs,^13^ evidence for elevated risks of chronic lymphocytic leukemia (CLL) and marginal zone lymphoma (MZL) remains limited.^14^ Nevertheless, in our previous work demonstrating shared genetic susceptibility between common IIM and B cell lymphoma subtypes, strong pleiotropic enrichments of SNPs with low *p* values for DM and PM when conditioned on CLL SNPs with *p* values of <0.001 were observed.^12^ Given the observed relationship between IIMs and B cell lymphomas, we cross-analyzed summary statistics from published genetic studies of IIM and four common B cell lymphoma subtypes, including DLBCL, FL, CLL, and MZL, to identify genetic variants associated with the risk of IIMs.

## Material and methods

### Data sources

Summary statistics of genome-wide SNP associations with DM and PM were obtained from the Genetics Scientific Interest Group (MYOGEN) of the International Myositis Assessment & Clinical Studies Group (IMACS). These summary-level data were derived from meta-analyses of prior GWASs^2^ and ImmunoChip studies,^3^ separately imputed to the TOPMed reference panel (version R2).^9^ Details of the imputation and quality control procedures have been described previously.^9^ In brief, variants were retained if they had imputation quality ≥0.6, minor allele frequency ≥0.005, and passed Hardy-Weinberg equilibrium testing in controls. No patients with IBM were included in the PM group of the GWAS and ImmunoChip studies.^2^^,^^3^ The DM and PM cohort comprised 1,131 and 1,094 individuals, respectively, and 11,697 individuals in the control group. Summary statistics for SNPs linked to the risks of DLBCL, FL, CLL, and MZL resulting from previous GWASs were obtained from the International Lymphoma Epidemiology Consortium (InterLymph).^15^^,^^16^^,^^17^^,^^18^ The CLL cohort included approximately 30% of individuals with small lymphocytic lymphoma, representing the same disease entity without peripheral blood lymphocytosis.^19^ Among B cell lymphoma subtypes, the group numbers were 3,587 (DLBCL), 2,847 (FL), 3,100 (CLL), and 825 (MZL), with control sample sizes ranging from approximately 6,000 to 8,000. All genetic data were obtained from individuals of European ancestry. Potential population stratification was accounted for in the original GWAS analyses.^2^^,^^3^^,^^9^^,^^15^^,^^16^^,^^17^^,^^18^ After additional quality control and standardization, summary statistics in the hg19 genome build for DM and PM from the meta-analysis study were paired to each B cell lymphoma subtype, resulting in a total of eight disease pairs (four per IIM subtype; see supplemental methods and Table S1).

### Statistical methods

To identify genetic variants associated with DM and PM risk, we applied conditional false discovery rate (condFDR)^20^ and multi-trait analysis of GWASs (MTAG),^21^ which leverage genetic correlation across traits to increase statistical power under distinct modeling frameworks. These approaches enable the detection of SNPs associated with IIM risk that may not have reached genome-wide significance (*p* value <5 × 10^−8^) in single-trait analyses due to limited power. Analyses were restricted to SNPs shared between each disease pair and aligned to the reference panel (Table S2).

CondFDR is a model-free method that recalibrates nominal *p* values for SNP associations with a primary phenotype by leveraging genetic pleiotropy with a secondary phenotype.^20^ We computed the condFDR of SNPs of a given IIM subtype based on the conditional empirical cumulative distribution function (cdf) of the *p* values of the corresponding SNPs of a given B cell lymphoma subtype. To mitigate false-positive findings, nominal *p* values of SNPs were adjusted using intergenic inflation control. SNPs within the HLA and chromosome 8p23.1 regions were excluded from fitting the conditional empirical cdf but retained for discovery analyses. A conservative significance threshold of condFDR <0.01 was applied.

MTAG exploits correlations in GWAS effect estimates arising from shared genetic architecture or correlated estimation errors across traits to improve statistical efficiency.^21^ We meta-analyzed summary statistics for DM and PM separately with those of the four B cell lymphoma subtypes, using a genome-wide significance threshold of *p* < 5 × 10^−8^. Because MTAG assumes a common variance-covariance structure of SNP effect sizes across traits, violation of this assumption may inflate type I error. This potential bias was assessed by computing the maximum FDR (maxFDR) under a multivariate spike-and-slab model.^21^ Given the substantial computational burden for more than two traits, we calculated pairwise maxFDR for each DM and PM disease pair and retained the maximum value.

Among SNPs reaching statistical significance in either analysis, we identified the lead SNPs through clumping (*r*^2^ < 0.1 in linkage disequilibrium [LD] windows of 10,000 kb and merged loci closer than 250 kb). To elucidate the functional relevance of the previously unreported non-HLA loci identified in our study, FUMA SNP2GENE (version 2.0.0) was used for positional mapping of lead SNPs and their high-LD proxies (*r*^2^ > 0.8). For these identified variants, we further performed *cis*-acting expression quantitative trait locus (*cis*-eQTL) analysis to identify variants regulating gene expression within a 1-Mb window and used Hi-C chromatin interaction mapping to detect long-range spatial contacts between genomic regions.^22^ The direction of eQTL effect was available only for variants included in our dataset. Parameter settings are described in Table S3. In brief, mappings considered only protein-coding genes, with significant eQTL association (FDR < 0.05) and chromatin interactions (FDR < 1 × 10^−6^, a recommended stringent threshold to ensure most confident Hi-C contacts^23^) retained. Chromatin interactions were further filtered to include only interactions between enhancers (regions that overlap with the tested SNP) and promoter regions of genes. Roadmap Epigenomics Projects for 111 epigenomes was used for enhancer and promoter annotations.^24^ All eQTL and Hi-C datasets used are of high quality and come from tissue or cell types relevant to IIMs.^22^

Gene Ontology (GO) enrichment analysis (biological process only) was performed separately for functionally mapped DM and PM risk-associated genes using clusterProfiler and the org.Hs.eg.db annotation packages.^25^^,^^26^ Significance was defined as an FDR of <0.05. All GO terms, regardless of statistical significance, were then simplified by semantic similarity-based clustering (threshold = 0.7) using rrvgo.^27^ Gene set enrichment analyses based on Kyoto Encyclopedia of Genes and Genomes (KEGG), Reactome, and WikiPathways were performed using FUMA GENE2FUNC (version 2.0.0), with significance defined as a Benjamini-Hochberg adjusted *p* value of <0.05.^22^

This study was approved by the Swedish Ethical Review Authority (2020-04529 and 2023-01343-02).

## Results

In the condFDR analyses, five loci each were identified for DM and PM with condFDR <0.01 when conditioning on the four B cell lymphoma subtypes (Tables 1, S4, S5, and S6). Of these, three (DM) and two (PM) represent candidate loci not previously reported at genome-wide significance. All lead SNPs reside in non-coding regions, predominantly intergenic. Except for SNP chr12:58674304T>C (DM), all lead variants had *p* values below the suggestive association threshold (1 × 10^−5^) in the IIM meta-analysis study.^9^ No identified loci were shared between DM and PM. Notably, variant chr13:110799415C>T, associated with DM risk, was identified across multiple conditioning analyses (DLBCL, CLL, and MZL), whereas the PM risk-associated variant chr4:971496T>C was consistently detected when conditioning on FL and CLL.

**Table 1:** Loci associated with dermatomyositis and polymyositis risk identified by conditioning on four common B cell lymphoma subtypes

Disease	Conditioning disease	SNP ID	chr:bp in hg19	Function	Reference allele	Effect allele	*Z* score	*p*value^a^	CondFDR	Nearest protein-coding genes^b^
Dermatomyositis	FL	rs117912338	12:58674304	intergenic	T	C	4.5289	1.78 × 10^−5^	0.0058	–
	DLBCL, CLL, MZL	rs2084040	13:110799415	intergenic	C	T	5.3744	3.51 × 10^−7^	0.0020	*COL4A1*
	MZL	rs8080734	17:38103285	intergenic	G	A	−4.7510	6.74 × 10^−6^	0.0094	*ORMDL3*, *LRRC3C*, *GSDMA*
Polymyositis	FL, CLL	rs116747058	4:971496	intergenic	T	C	5.0740	3.51 × 10^−6^	0.0045	*DGKQ*, *SLC26A1*, *IDUA*, *GAK*
	CLL	rs12203592	6:396321	intronic	C	T	5.1260	2.78 × 10^−6^	0.0002	***IRF4***

SNP, single-nucleotide polymorphism; DLBCL, diffuse large B cell lymphoma; FL, follicular lymphoma; CLL, chronic lymphocytic leukemia; MZL, marginal zone lymphoma; chr:bp, chromosome:basepairs; CondFDR, conditional false discovery rate.

a The *p* values were adjusted using intergenic inflation control.

b The lead SNPs and non-GWAS-tagged candidate SNPs in linkage disequilibrium (*r*^2^ > 0.8) with the lead SNPs were mapped in this positional mapping. The gene containing an intronic lead SNP is highlighted in bold.

In the MTAG analyses, all genome-wide significant SNPs associated with DM risk were located within the HLA region and corresponded to previously reported loci. For PM, a previously unreported locus reaching genome-wide significance (lead SNP chr6:32650631C>A, intergenic; *p* value = 1.44 × 10^−8^) within the HLA class II region was identified, with a maxFDR of 0.0003.

### Functional annotation for DM-associated variants

**Figure 1:**
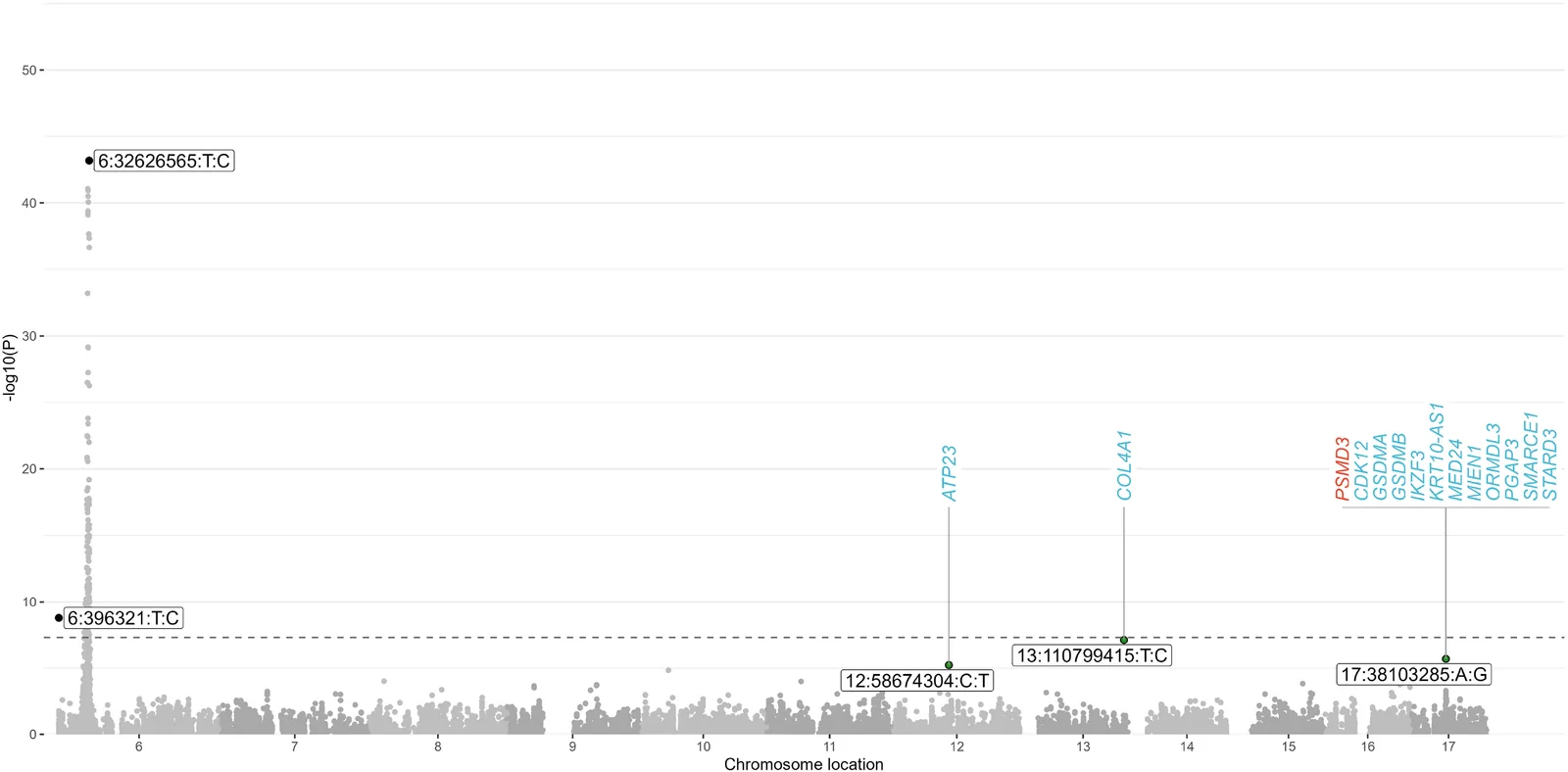
Manhattan plot of dermatomyositis risk-associated loci identified in the conditional false discovery rate (condFDR) analysis (condFDR < 0.01), with functionally mapped genes annotated for previously unreported loci Genes supported by eQTL evidence only are shown in blue, while those supported by both eQTL and Hi-C evidence are shown in red. The *x* axis of the plot is truncated to focus on chromosomes with statistically significant signals. eQTL, expression quantitative trait locus.

All identified DM risk-associated lead SNPs function as *cis*-eQTLs in IIM-relevant tissues/cell types, with the direction of effect specified (Figure 1 and Table S7). The variant chr12:58674304T>C downregulates *ATP23* (>320 kb upstream; MIM: 619760), while an LD proxy of chr13:110799415C>T regulates the nearby gene *COL4A1* (MIM:120130). The variant chr17:38103285G>A shows complex regulatory effects: it upregulates *CDK12* (MIM: 615514) and *MIEN1* (MIM: 611802); downregulates *KRT10-AS1*, *PSMD3* (MIM: 617676), *SMARCE1* (MIM: 603111), and *STARD3* (MIM: 607048); and bidirectionally regulates *PGAP3* (MIM: 611801), *GSDMA* (MIM: 611218), *GSDMB* (MIM: 611221), *IKZF3* (MIM: 606221), *MED24* (MIM: 607000), and *ORMDL3* (MIM: 610075). Beyond blood, regulatory effects were also observed in fibroblasts, gastrointestinal tissues, and adipose tissue, affecting genes such as *GSDMA* (MIM: 611218), *GSDMB* (MIM: 611221), *MED24* (MIM: 607000), and *ORMDL3* (MIM: 610075). Hi-C chromatin interaction mapping further linked chr17:38103285G>A to *PSMD3* (MIM: 617676) in brain tissue (Figure 1 and Table S8).

The 14 genes mapped via eQTL associations or chromatin interactions for the three DM risk loci and their LD proxies were annotated to 253 GO biological processes (Table S9). Many of these processes were related to lipid metabolic and biosynthetic processes and immune regulation, although none showed significant enrichment. No significant results were identified in KEGG, Reactome, or WikiPathways enrichment analyses.

### Functional annotation for PM-associated variants

**Figure 2:**
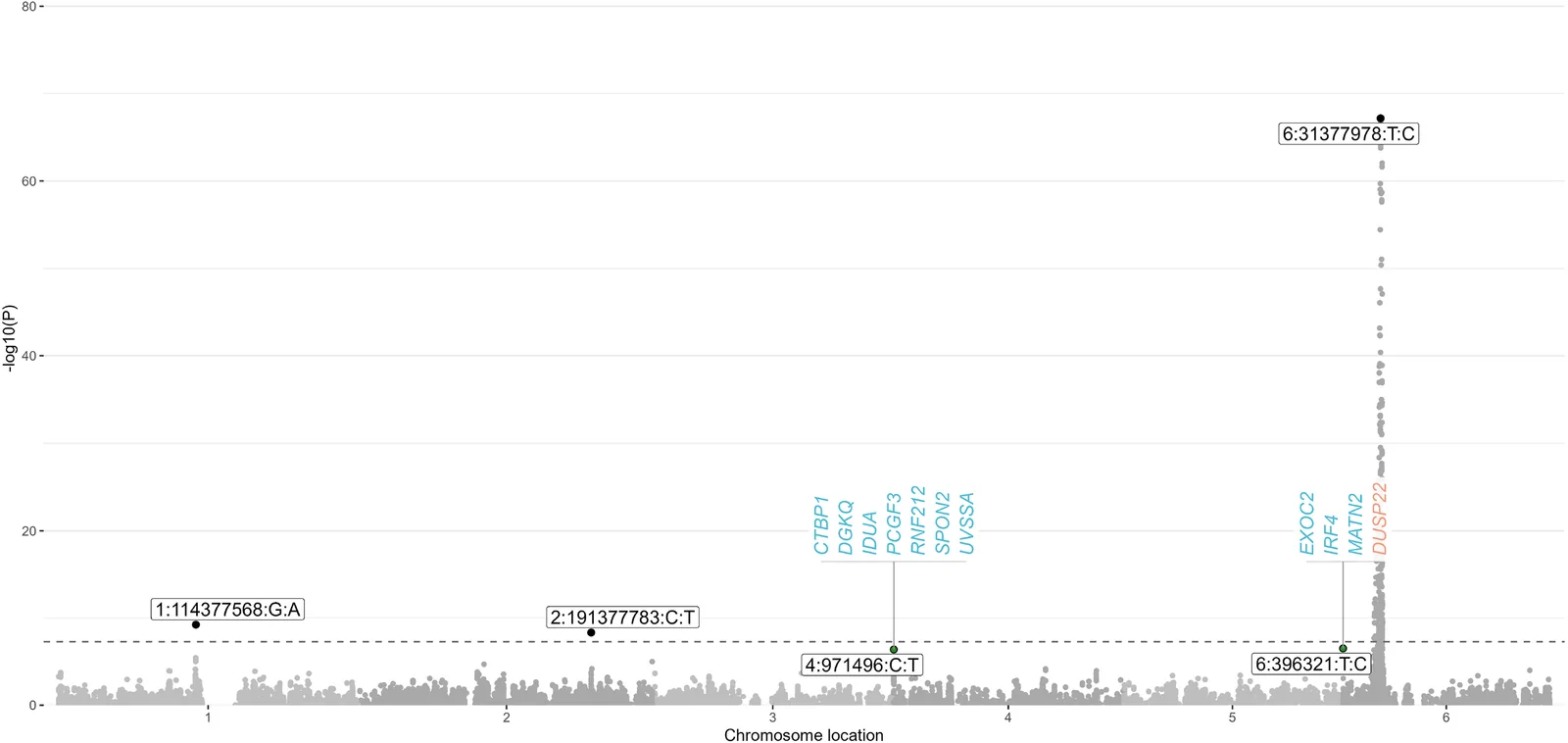
Manhattan plot of polymyositis risk-associated loci identified in the condFDR analysis (condFDR < 0.01), with functionally mapped genes annotated for previously unreported loci Genes supported by eQTL evidence only are shown in blue, and those supported by Hi-C evidence only in coral. The *x* axis of the plot is truncated to focus on chromosomes with statistically significant signals. eQTL, expression quantitative trait locus.

The two PM risk-associated lead SNPs function as *cis*-eQTLs in blood (Figure 2 and Table S10). The variant chr4:971496T>C was associated with regulation of multiple genes, including upregulation of *CTBP1* (MIM: 602618), *IDUA* (MIM: 252800), and *SPON2* (MIM: 605918); downregulation of *PCGF3* (MIM: 617543), *RNF212* (MIM: 612041), and *UVSSA* (MIM: 614632); and bidirectional regulation of *DGKQ* (MIM: 601207). The variant chr6:396321C>T, previously linked to IIM overall, DM, and juvenile DM,^9^ was here associated with PM and shows *cis*-eQTL effects on *EXOC2* (MIM: 615329) and *MATN2* (MIM: 602108) in addition to *IRF4* (MIM: 601900). Chromatin interaction analyses further linked this variant to *DUSP22* (MIM: 616778) in spleen and lymphoblastoid cells (Figure 2 and Table S11).

Eleven genes identified through functional mapping for the two PM risk loci were annotated to 394 GO biological processes, with four synaptic vesicle cycle (SVC) processes significantly enriched (gene set: *GAK* [MIM: 602052], *DGKQ* [MIM: 601207], and *CTBP1* [MIM: 602618]; fold enrichment: 57.3; adjusted *p* value = 0.003) (Table S12). Although they lack statistical significance, additional biological processes related to lipid metabolism and biosynthesis, and immune regulation, were also annotated. No significant enrichment was observed in KEGG, Reactome, or WikiPathways.

## Discussion

In this study, we improved statistical efficiency in detecting genetic associations with IIM risk by using condFDR and MTAG, leveraging genetic correlation with B cell lymphomas. A major challenge in IIM genetics is the limited cohort size for identifying common variants with moderate effect sizes. Although prior epidemiological studies found no statistically significant familial associations between IIMs and B cell lymphomas (standardized incidence ratio/adjusted odds ratio ranging from 1.2 to 2.0),^28^^,^^29^ we previously demonstrated locus-specific genetic correlations, primarily in the HLA region and several non-HLA regions.^12^ Consistent with these findings, we identified a previously unreported HLA class II locus associated with PM at genome-wide significance (chr6:32650631C>A); however, the corresponding HLA allele and amino acid could not be determined due to the lack of HLA imputation data. The *cis*-eQTL chr6:396321C>T identified for PM, which has also reached genome-wide significance for DM and juvenile DM,^9^ was previously shown to be jointly associated with CLL for both DM and PM.^12^ Earlier ImmunoChip-based analyses also suggested genetic correlations at regions chr1:205917549–208162951 (DM and DLBCL) and chr9:138995792–140097759 (PM and DLBCL). However, no SNP associations were identified within these regions in the present study. Nevertheless, genetic correlations between IIMs and B cell lymphomas may have been underestimated in previous analyses, as methods for estimating locus-specific genetic correlation generally have limited statistical power for binary traits.^30^

We replicated several previously reported non-HLA genetic associations for DM, PM, and IIM, including *GSDMB* (MIM: 611221), *DGKQ* (MIM: 601207), *SLC26A1* (MIM: 610130), and *IDUA* (MIM: 252800).^3^^,^^6^^,^^7^ The *GSDMB* (MIM: 611221) eQTL variant chr17:38066267G>T, previously reported with suggestive significance for DM,^3^ is in moderate LD (*r*^2^ = 0.5) with our identified *GSDMB* (MIM: 611221) eQTL variant chr17:38103285G>A in European populations. For *DGKQ* (MIM: 601207), several eQTLs have been previously reported for IIMs.^3^^,^^6^^,^^7^ Moreover, an intronic *SLC26A1* (MIM: 610130)/*IDUA* (MIM: 252800) variant has been linked to PM risk without a known functional role.^3^ Here, we identified a previously unreported independent variant (chr4:971496T>C) acting as a *cis*-eQTL for *DGKQ* (MIM: 601207) and *IDUA* (MIM: 252800) in immune-related cells. These regulatory polymorphisms further support the involvement of these genes in IIM pathogenesis.

The PM risk-associated variant chr4:971496T>C was linked to genes involved in the SVC, including *DGKQ* (MIM: 601207), *CTBP1* (MIM: 602618), and *GAK* (MIM: 602052). Dysfunction of this pathway has been implicated in the accumulation of neurodegenerative proteins such as amyloid-beta and tau, pathological hallmarks shared between IBM skeletal muscles and Alzheimer disease brain tissue.^1^^,^^31^ However, SVC-related mechanisms have not previously been implicated in PM or other non-DM IIM subtypes, warranting further investigation into their potential role in PM pathogenesis.

Another intriguing finding was the identification of variant mapping to genes involved in biological processes implicated in IIM pathogenesis. Several DM- and PM-associated genes participate in immune-related activities, including T cell and B cell regulation (DM: *IKZF3* [MIM: 606221] and *ORMDL3* [MIM: 610075]; PM: *IRF4* [MIM: 601900] and *DUSP22* [MIM: 616778]) and cytokine production (PM: *SPON2* [MIM: 605918]), key components of IIM pathophysiology, and therapeutic targets.^32^ Additionally, DM-associated genes *ATP23* (MIM: 619760) and *PSMD3* (MIM: 617676) are linked to protein homeostasis. *PSMD3* (MIM: 617676), supported by both eQTL and chromatin interaction evidence, encodes a 26S proteasome regulatory subunit involved in the ubiquitin-proteasome pathway. These findings align with prior reports of dysregulated proteosome- and ubiquitination-related protein expression in peripheral blood of patients with DM.^33^ Furthermore, a protein-protein interaction and pathway analysis of IIM-related autoantigens and proteins encoded by IIM risk-associated loci revealed direct interactions among ubiquitin proteasome pathway proteins, including PSMD3.^34^

We also identified DM risk-associated genes *PGAP3* (MIM: 611801), *ORMDL3* (MIM: 610075), and *STARD3* (MIM: 607048), and the PM risk-associated gene *DGKQ* (MIM: 601207), related to lipid metabolism and biosynthesis, supporting accumulating evidence of lipid dysregulation across IIM subtypes.^35^^,^^36^^,^^37^^,^^38^^,^^39^

Notably, *COL4A1* (MIM: 120130), encoding the collagen type IV alpha 1 chain, a key structural component of the basement membrane, was identified as a DM risk-associated gene. Pathogenic variants in *COL4A1* (MIM: 120130) cause a multi-organ disorder characterized by hereditary angiopathy, nephropathy, aneurysms, and muscle cramps.^40^ Consistently, *Col4a1* mutant mouse models exhibit progressive skeletal muscle myopathy, potentially due to vascular defects.^41^^,^^42^ These findings suggest a potential link between DM and COL4A1-related myopathy, which requires further investigation.

The previously unreported non-HLA loci identified via condFDR were not replicated in the MTAG analyses, likely reflecting lower statistical power gain with MTAG in this context. MTAG performance depends on the genetic covariance across traits,^43^ and as shared genetic susceptibility between IIMs and B cell lymphomas is primarily found within the HLA region,^12^ gains for non-HLA locus discovery were limited.

This study has several limitations. First, independent replication was not feasible due to the rarity and heterogeneity of IIMs, and additional functional validation (e.g., fine-mapping and colocalization) was constrained by the limited statistical power of the original DM and PM meta-analyses. Future validation will require larger cohorts through international collaboration. Second, PM in the MYOGEN dataset was defined using the Bohan and Peter criteria, potentially including non-DM IIM subtypes such as IMNM and ASyS. While IBM and anti-Jo1-positive cases were excluded where applicable,^2^^,^^3^ the identified PM risk-associated variants may reflect combined effects across PM, IMNM, ASyS, and overlap myositis. Finally, as the analyses were restricted to individuals of European ancestry, our findings may not generalize to other populations.

Despite these limitations, the application of power-enhancing analytical approaches combined with functional annotation enabled the identification of five previously unreported regulatory variants (three for DM and two for PM). These variants affect genes involved in SVC pathways, immune regulation, protein homeostasis, lipid metabolism and biosynthesis, and myopathy. Collectively, these findings advance the understanding of the genetic architecture underlying IIM pathogenesis and are useful for guiding future genetic studies.

## Data and code availability

Due to data-use agreements with the International Myositis Assessment & Clinical Studies Group and the International Lymphoma Epidemiology Consortium, the summary statistics used in this study are not publicly available and can only be accessed with approval from the respective consortia. This study did not generate any new code.

## Acknowledgments

This work was supported by the 10.13039/501100004359Swedish Research Council, the 10.13039/100012538Swedish Cancer Foundation, the UM Macao Fellow scheme of the UM Talent Program of the University of Macau (file no. UMTP2023-MF02), and R01 AR078785 from the 10.13039/100000002National Institutes of Health/10.13039/100000069National Institute of Arthritis and Musculoskeletal and Skin Diseases (J.N.J.). We would like to thank Lina Marcela Diaz-Gallo for outstanding advice on study design. We also want to acknowledge IMACS, the MYOGEN members, the LM GWAS Coordinating Committee, and the InterLymph Data Coordinating Committee for their indispensable support in data application and transfer and for critical review of the manuscript. Special thanks are extended to Arial Harrison, Dakai Zhu, and Dennis Robinson for assisting the data transfer. The contributing members of the IMACS MYOGEN and InterLymph Consortium are listed in the supplemental appendices.

## Author contributions

All authors conceived and designed the work; W.I.C. curated and standardized the GWAS summary statistics, performed all analyses, interpreted the findings, and drafted the manuscript; and all authors contributed to revising the manuscript critically and intellectual content.

## Declaration of interests

I.E.L. and M.H. have received grants from Swedish Research Council.

## Web resources

OMIM, https://www.omim.org/

## Supplemental information

Document S1. Supplemental methods, Figures S1 and S2, Tables S1–S4, and Appendices S1–S3

Data S1. Tables S5–S12

Document S2. Article plus supplemental information
