# Supplementary material for: Identification of genetic variants associated with idiopathic inflammatory myopathies via cross-trait analysis with B cell lymphomas
Source: HGG Adv. 2026 Jul 24;7(4):100658. doi: 10.1016/j.xhgg.2026.100658 (PMC13495596; doi:10.1016/j.xhgg.2026.100658)
Supplement: Supplementary file 1 [file mmc1.pdf]

HGGA, Volume 7

Supplemental information

Identification of genetic variants associated with idiopathic inflammatory myopathies via cross-trait analysis with B cell lymphomas

Weng Ian Che, James N. Jarvis, Anton Öberg Sysojev, Catherine Zhu, Karina Patasova, International Lymphoma Epidemiology Consortium (InterLymph), IMACS Genetics Group (MYOGEN), Karin E. Smedby, Ingrid E. Lundberg, Helga Westerlind, Janine A. Lamb, and Marie Holmqvist

Identification of genetic variants associated with idiopathic inflammatory myopathies via cross-trait analysis with B-cell lymphomas

Weng Ian Che[1], James N. Jarvis[2], Anton Öberg Sysojev[3], Catherine Zhu[4], Karina Patasova[3], International Lymphoma Epidemiology Consortium (InterLymph)#, IMACS Genetics Group (MYOGEN)#, Karin E. Smedby[3, 5], Ingrid E. Lundberg[6, 7], Helga Westerlind[3], Janine A Lamb*[8], Marie Holmqvist*[3, 7]

[1]Department of Public Health and Medicinal Administration, Faculty of Health Sciences, University of Macau, Macau SAR, China. [2]Department of Pediatrics and Center for Indigenous Health, University of Washington School of Medicine, Seattle, WA, USA. [3]Clinical epidemiology division, Department of Medicine, Solna, Karolinska Institutet, Stockholm, Sweden. [4]Institute for Clinical and Translational Research, Baylor College of Medicine, Houston, TX, USA. [5]Hematology Center, Karolinska University Hospital, Stockholm, Sweden. [6]Division of rheumatology, Department of Medicine, Solna, Karolinska Institutet, Stockholm, Sweden. [7]ME Gastro, Derm and Rheuma, Theme Inflammation and Aging, Karolinska University Hospital, Stockholm, Sweden. [8]Epidemiology and Public Health Group, School of Health Sciences, The University of Manchester, Manchester, UK.

*Dr. Janine A Lamb and Dr. Marie Holmqvist contributed equally to this work.

#The contributing members of the disease consortia are listed in the supplementary appendices.

Corresponding author

Marie Holmqvist

Address: Department of Medicine, Solna, Eugeniahemmet, T2, Karolinska Universitetssjukhuset Solna 171 76 Stockholm, Sweden

Email: marie.holmqvist@ki.se

ORCiD: https://orcid.org/0000-0001-8996-5260

Supplementary materials

SUPPLEMENTARY METHODS

Quality control and standardization

Summary statistics data of IIM and B-cell lymphoma subtypes were quality controlled and standardized. For the MYOGEN data, we filtered out SNPs that were multiallelic or located in conversion unstable regions (defined according to the published list of conversion unstable regions in GRCh38[1]). We lifted SNPs from the MYOGEN data to GRCh37 from GRCh38 using rtracklayer::liftOver in R[2,3] and SNPs that failed to convert were excluded. For the InterLymph data, we filtered duplicated SNPs and SNPs with association testing computed from a single study group. We used the R package MungeSumstats to standardise summary statistics from the MYOGEN and InterLymph consortia and computed Z scores of SNPs based on p-values.[4,5] In the standardisation, reference alleles were aligned to the 1000 Genomes phase 2 Reference Genome Sequence in GRCh37. Z scores were flipped if effective alleles rather than the reference alleles were aligned. SNPs that were ambiguous, unmatched to or missing on the reference genome, or with null effect size were filtered.

SUPPLEMENTARY TABLES

Table S1. Number of cases, controls, and single nucleotide polymorphisms (SNPs) included after in quality control and data standardization

		MYOGEN		InterLymph			
	**Number of SNPs/patients**	**Meta-analysis DM**	**Meta-analysis PM**	**DLBCL**	**FL**	**CLL**	**MZL**
**Cases**		1,131	1,094	3,587	2,847	3,100	825
**Controls**		11,697	11,697	7,666	8,107	7,667	6,221
	**Imputed SNPs**	1,255,706	1,254,738	9,116,853	9,078,855	9,098,434	8,478,065
**Stable biallelic SNPs**	Single-study group	-	-	498,560	460,723	487,417	-
	SNP duplicates	-	-	2	8	3	-
	Missing OR	-	-	-	-	-	-
	Multiallelic	83,756	83,692	-	-	-	-
**LiftOver to GRCh37**	Conversion unstable SNPs	50	50	-	-	-	-
	Failed to be converted	836	836	-	-	-	-
	Missing on reference genome	74	74	1,228	1,230	1,230	1,212
**QC and Alignment**	Ambiguous SNPs	177,921	177,751	1,330,560	1,330,713	1,329,637	1,309,967
	Zero effect value	-	-	6,773	6,252	6,398	3,776
	Not matched to reference genome	69	69	-	-	-	-
**Qced SNPs**	**After QC and alignment**	**993,000**	**992,266**	**7,279,730**	**7,279,929**	**7,273,749**	**7,163,110**

DM: Dermatomyositis; PM: Polymyositis; DLBCL: Diffuse large B-cell lymphoma; FL: Follicular lymphoma; CLL: Chronic lymphocytic leukaemia; MZL: Marginal zone lymphoma.

Table S2. Number of single nucleotide polymorphisms for each disease pair included in the conditional false discovery rate (condFDR) and multi-trait association of GWAS (MTAG) analyses

	Number of SNPs analysed	
	**CondFDR**	**MTAG**
**DM-DLBCL**	833,376	889,442
**DM-FL**	833,458	889,442
**DM-CLL**	833,318	889,442
**DM-MZL**	831,324	889,442
**PM-DLBCL**	833,281	889,198
**PM-FL**	833,377	889,198
**PM-CLL**	833,240	889,198
**PM-MZL**	831,242	889,198

GWAS: Genome-wide association study; DM: Dermatomyositis; PM: Polymyositis; DLBCL: Diffuse large B-cell lymphoma; FL: Follicular lymphoma; CLL: Chronic lymphocytic leukaemia; MZL: Marginal zone lymphoma.

Table S3. Parameter settings in FUMA SNP2GENE

**Parameters for candidate SNPs identification for all identified SNPs**	
r2	0.8
Reference panel	1KG/Phase3 EUR
Include variants in reference panel (non-GWAS tagged SNPs in linkage disequilibrium, LD)	Yes
Maximum distance between LD blocks to merge into a locus (< kb)	250
	Positional mapping
Maximum distance to genes (kb)	10
CADD, Regulome or 15-core chromatin state filtering	No
**eQTL mapping for newly identified SNPs**	
Tissue type	eQTLcatalogue/Alasoo_2018_ge_macrophage_IFNg_Salmonella.txt.gz, eQTLcatalogue/Alasoo_2018_ge_macrophage_IFNg.txt.gz, eQTLcatalogue/Alasoo_2018_ge_macrophage_naive.txt.gz, eQTLcatalogue/Alasoo_2018_ge_macrophage_Salmonella.txt.gz, eQTLcatalogue/BLUEPRINT_ge_monocyte.txt.gz, eQTLcatalogue/BLUEPRINT_ge_neutrophil.txt.gz, eQTLcatalogue/BLUEPRINT_ge_T-cell.txt.gz, eQTLcatalogue/BrainSeq_ge_brain.txt.gz, eQTLcatalogue/CEDAR_B-cell_CD19.txt.gz, eQTLcatalogue/CEDAR_ileum.txt.gz, eQTLcatalogue/CEDAR_monocyte_CD14.txt.gz, eQTLcatalogue/CEDAR_neutrophil_CD15.txt.gz, eQTLcatalogue/CEDAR_platelet.txt.gz, eQTLcatalogue/CEDAR_rectum.txt.gz, eQTLcatalogue/CEDAR_T-cell_CD4.txt.gz, eQTLcatalogue/CEDAR_T-cell_CD8.txt.gz, eQTLcatalogue/CEDAR_transverse_colon.txt.gz, eQTLcatalogue/Fairfax_2012_B-cell_CD19.txt.gz, eQTLcatalogue/Fairfax_2014_IFN24.txt.gz, eQTLcatalogue/Fairfax_2014_LPS24.txt.gz, eQTLcatalogue/Fairfax_2014_LPS2.txt.gz, eQTLcatalogue/Fairfax_2014_naive.txt.gz, eQTLcatalogue/GENCORD_ge_fibroblast.txt.gz, eQTLcatalogue/GENCORD_ge_LCL.txt.gz,

eQTLcatalogue/GENCORD_ge_T-cell.txt.gz, eQTLcatalogue/GEUVADIS_ge_LCL.txt.gz, eQTLcatalogue/HipSci_ge_iPSC.txt.gz, eQTLcatalogue/Kasela_2017_T-cell_CD4.txt.gz, eQTLcatalogue/Kasela_2017_T-cell_CD8.txt.gz, eQTLcatalogue/Lepik_2017_ge_blood.txt.gz, eQTLcatalogue/Naranbhai_2015_neutrophil_CD16.txt.gz, eQTLcatalogue/Nedelec_2016_ge_macrophage_Listeria.txt.gz, eQTLcatalogue/Nedelec_2016_ge_macrophage_naive.txt.gz, eQTLcatalogue/Nedelec_2016_ge_macrophage_Salmonella.txt.gz, eQTLcatalogue/Quach_2016_ge_monocyte_IAV.txt.gz, eQTLcatalogue/Quach_2016_ge_monocyte_LPS.txt.gz, eQTLcatalogue/Quach_2016_ge_monocyte_naive.txt.gz, eQTLcatalogue/Quach_2016_ge_monocyte_Pam3CSK4.txt.gz, eQTLcatalogue/Quach_2016_ge_monocyte_R848.txt.gz, eQTLcatalogue/Schwartzentruber_2018_ge_sensory_neuron.txt.gz, eQTLcatalogue/TwinsUK_ge_blood.txt.gz, eQTLcatalogue/TwinsUK_ge_fat.txt.gz, eQTLcatalogue/TwinsUK_ge_LCL.txt.gz, eQTLcatalogue/TwinsUK_ge_skin.txt.gz, eQTLcatalogue/van_de_Bunt_2015_ge_pancreatic_islet.txt.gz, scRNA_eQTLs/B_cell.txt.gz, scRNA_eQTLs/DC.txt.gz, scRNA_eQTLs/NK.txt.gz, scRNA_eQTLs/Monocyte.txt.gz, scRNA_eQTLs/Classical_Monocyte.txt.gz, scRNA_eQTLs/Non_classical_Monocyte.txt.gz, scRNA_eQTLs/T_CD4.txt.gz, scRNA_eQTLs/T_CD8.txt.gz, scRNA_eQTLs/PBMC.txt.gz, DICE/B_cell_naive.txt.gz, DICE/T_CD4_naive.txt.gz, DICE/T_CD4_naive_activated.txt.gz, DICE/T_CD8_naive.txt.gz, DICE/T_CD8_naive_activated.txt.gz, DICE/Monocyte_classical.txt.gz, DICE/Monocyte_non_classical.txt.gz, DICE/NK.txt.gz, DICE/T_CD4_TFH.txt.gz, DICE/T_CD4_TH1.txt.gz, DICE/T_CD4_TH17.txt.gz, DICE/T_CD4_TH1_17.txt.gz, DICE/T_CD4_TH2.txt.gz, DICE/T_CD4_memory_TREG.txt.gz, DICE/T_CD4_naive_TREG.txt.gz, eQTLGen/eQTLGen_cis_eQTLs.txt.gz, eQTLGen/eQTLGen_trans_eQTLs.txt.gz, GTEx/v8/Cells_EBV-transformed_lymphocytes.txt.gz, GTEx/v8/Whole_Blood.txt.gz, GTEx/v8/Colon_Sigmoid.txt.gz, GTEx/v8/Colon_Transverse.txt.gz, GTEx/v8/Esophagus_Gastroesophageal_Junction.txt.gz, GTEx/v8/Esophagus_Mucosa.txt.gz, GTEx/v8/Esophagus_Muscularis.txt.gz, GTEx/v8/Lung.txt.gz,

	GTEx/v8/Muscle_Skeletal.txt.gz, GTEx/v8/Cells_Cultured_fibroblasts.txt.gz, GTEx/v8/Skin_Not_Sun_Exposed_Suprapubic.txt.gz, GTEx/v8/Skin_Sun_Exposed_Lower_leg.txt.gz, GTEx/v8/Small_Intestine_Terminal_Ileum.txt.gz, GTEx/v8/Spleen.txt.gz
P-value threshold	FDR < 0.05
CADD, Regulome or 15-core chromatin state filtering	No
**3D Chromatin interaction mapping for newly identified SNPs**	
Builtin chromatin interaction data	EP/PsychENCODE/EP_links_oneway.txt.gz, HiC/PsychENCODE/Promoter_anchored_loops.txt.gz, HiC/GSE87112/Lung.txt.gz, HiC/GSE87112/Small_Bowel.txt.gz, HiC/GSE87112/Spleen.txt.gz, HiC/GSE87112/GM12878.txt.gz
P-value threshold	FDR $< 1 \times 10^{-6}$
Promoter region window	250bp upstream and 500bp downstream from transcriptional start site
Annotate enhancer/promoter regions (Roadmap 111 epigenomes)	E029, E030, E031, E032, E033, E034, E035, E036, E037, E038, E039, E040, E041, E042, E043, E044, E045, E046, E047, E048, E050, E051, E062, E075, E076, E106, E077, E078, E079, E084, E085, E109, E101, E102, E103, E092, E094, E110, E111, E017, E088, E096, E052, E089, E100, E107, E108, E055, E056, E057, E058, E059, E061, E113, E093, E112
Filter SNPs by enhancers	Yes
Filter genes by promoters	Yes
CADD, Regulome or 15-core chromatin state filtering	No
**Gene types**	
Ensemble version	v102
Gene type	protein_coding
**Others**	
Major histocompatibility complex exclusion	Yes for novel SNPs in all mappings

Table S4. Loci associated with dermatomyositis and polymyositis risk identified by conditioning on four common B-cell lymphoma subtypes, at genome-wide significance reported in previous studies

Disease	Conditioning disease	SNP ID	CHR:BP in hg19	Function	Reference allele	Effect allele	Z score	P-value§	Condfdr	Nearest protein coding genes#
**DM**	DLBCL, FL, CLL, MZL	rs12203592	6:396321	intronic	C	T	6.03	1.13E-08	7.41E-07	***IRF4***
	DLBCL, FL, CLL, MZL	rs9273371	6:32626565	upstream	C	T	13.90	1.31E-39	7.26E-29	*HLA-DQB1*
**PM**	DLBCL, FL, CLL, MZL	rs2476601	1:114377568	exonic	A	G	-6.20	1.44E-08	1.43E-04	***PTPN22****, PHTF1, RSBN1*
	DLBCL, FL, CLL, MZL	rs74925618	2:191377783	intronic	T	C	5.86	8.19E-08	4.34E-04	***NEMP2****, HIBCH, INPP1, MFSD6*
	DLBCL, FL, CLL, MZL	rs2523495	6:31377978	intronic	C	T	17.41	4.47E-57	4.82E-29	***MICA***

SNP: Single nucleotide polymorphism; GWAS: Genome-wide association study; DLBCL: Diffuse large B-cell lymphoma; FL: Follicular lymphoma; CLL: Chronic lymphocytic leukaemia; MZL: Marginal zone lymphoma; CHR:BP: Chromosome:Base pair; condfdr: Conditional false discovery rate

§ The p-values were adjusted using intergenic inflation control.

#The lead SNPs and non-GWAS-tagged candidate SNPs in linkage disequilibrium ($r^2 \geq 0.8$) with the lead SNPs were mapped in this positional mapping. The gene containing a lead SNP is highlighted in bold.

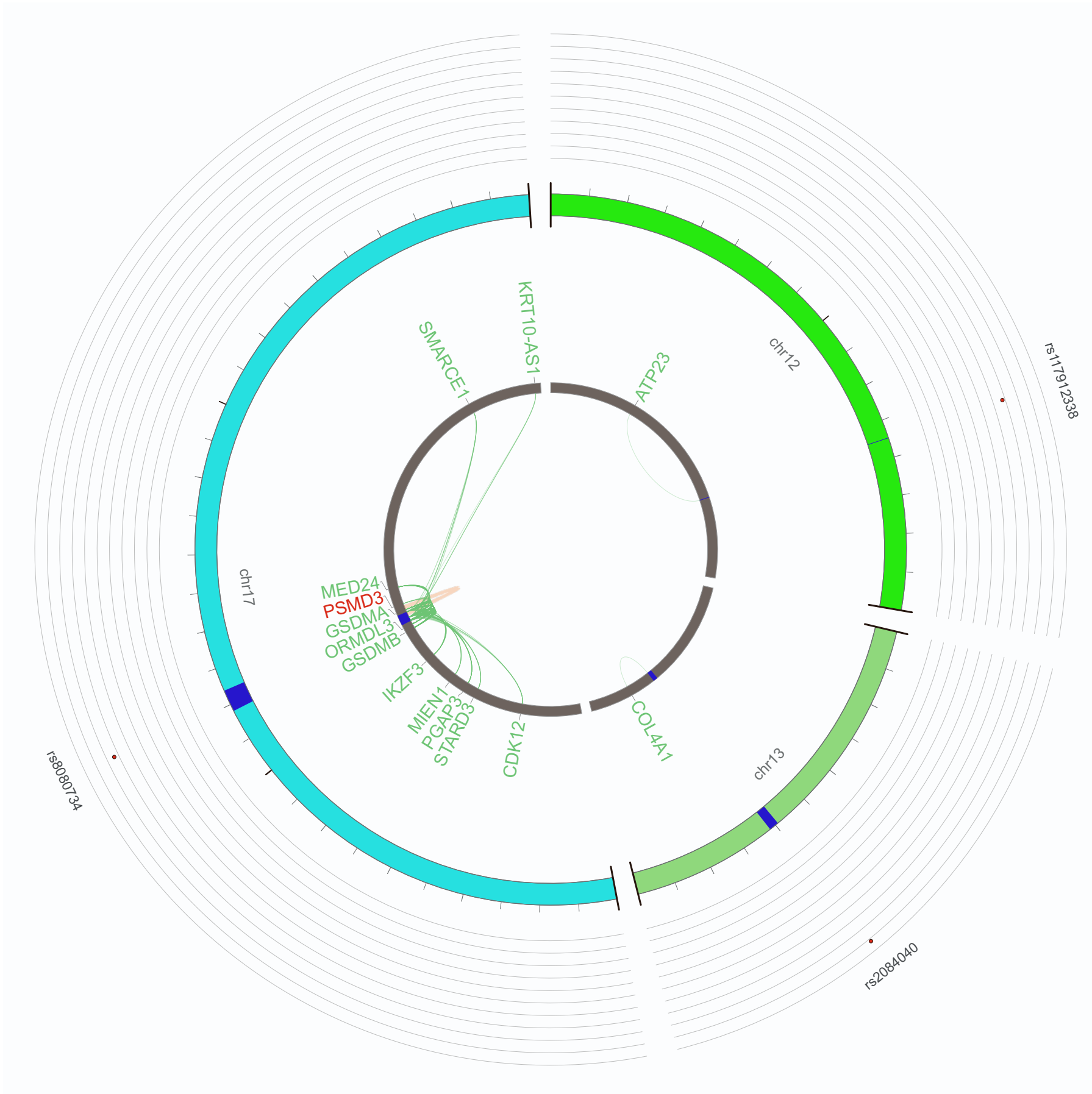

Supplementary Figure S1. eQTLs and Hi-C chromatin interactions at novel DM risk loci. Data is presented for risk loci on chromosomes 12, 13, and 17, arranged clockwise. The outermost outer layer is the Manhattan plot displaying the lead SNPs with SNP ID labelled. The outer circle is the chromosome coordinate with genomic risk loci are highlighted in blue. Genes mapped by either eQTLs (green), chromatin interaction (orange) or both (red) are shown on the inner circle. eQTL and chromatin interaction links are shown as links in green and orange, respectively. DM: Dermatomyositis; SNP: Single nucleotide polymorphism; eQTL: expression quantitative trait locus.

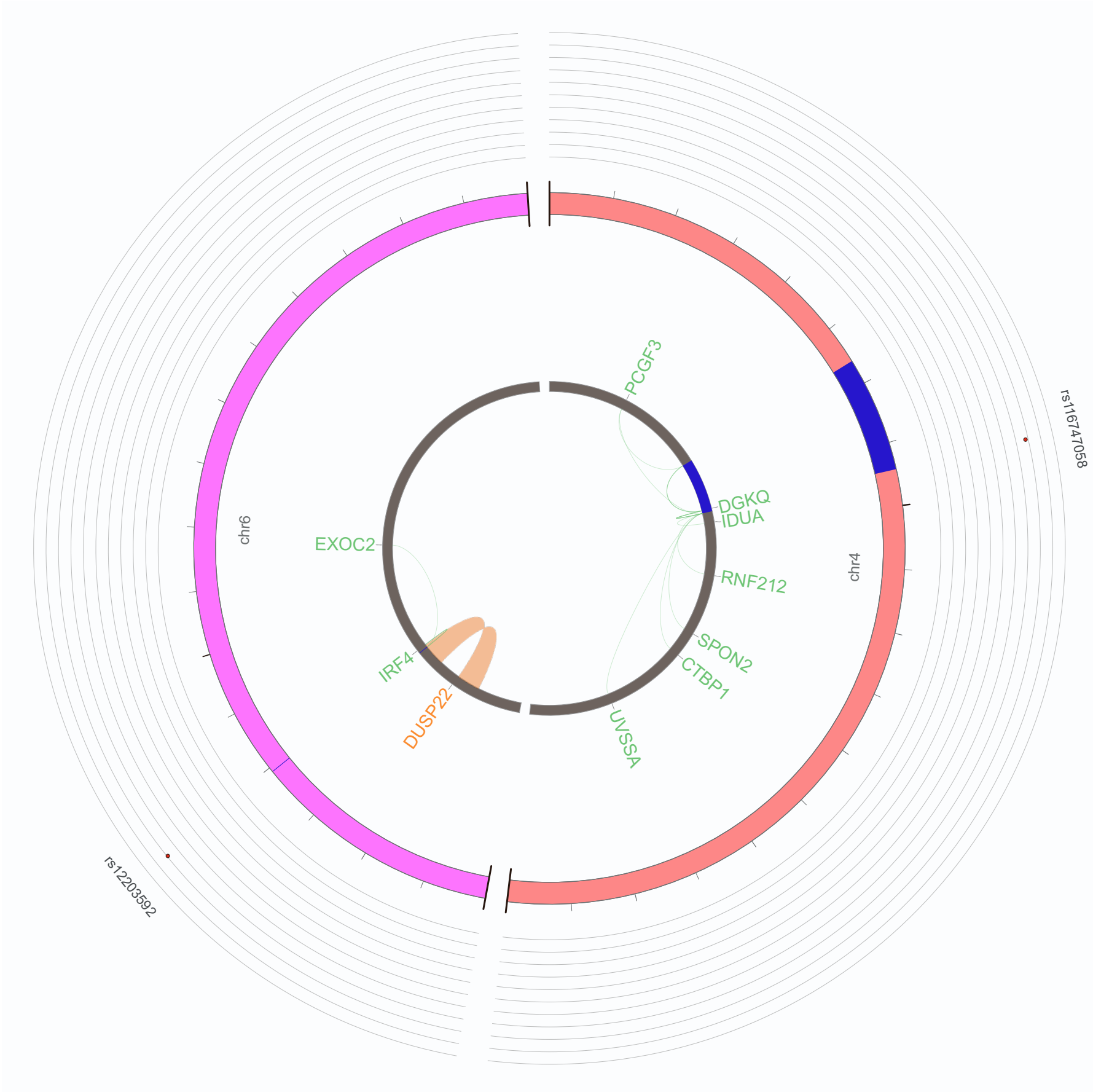

Supplementary Figure S2. eQTLs and Hi-C chromatin interactions at novel PM risk loci. Data is presented for risk loci on chromosomes 4, and 6, arranged clockwise. The outermost layer is the Manhattan plot displaying the lead SNPs with SNP ID labelled. The outer circle is the chromosome coordinate with genomic risk loci are highlighted in blue. Genes mapped by either eQTLs (green) or chromatin interaction (orange) are shown on the inner circle. eQTL and chromatin interaction links are shown as links in green and orange, respectively. PM: Polymyositis; SNP: Single nucleotide polymorphism; eQTL: expression quantitative trait locus.

SUPPLEMENTARY APPENDIX I: Investigators from IMACS Myositis Genetics Scientific Interest Group (MYOGEN) contributing to MYOGEN GWAS and ImmunoChip studies

Chris Amos. Baylor College of Medicine, Houston, Texas.

Olivier Benveniste. Department of Internal Medicine and Clinical Immunology, Pitié-Salpêtrière Hospital, Paris, France.

Hector Chinoy. National Institute for Health Research Manchester Biomedical Research Centre, Manchester University NHS Foundation Trust, The University of Manchester, Manchester, UK, and Department of Rheumatology, Salford Royal Hospital, Northern Care Alliance NHS Foundation Trust, Manchester Academic Health Science Centre, Salford, UK, and Centre for Musculoskeletal Research, Faculty of Biology, Medicine and Health, The University of Manchester, Manchester, UK.

Jan L De Bleecker, Boel De Paepe. Department of Neurology, Ghent University, Ghent, Belgium.

Andrea Doria. Rheumatology Unit, Department of Medicine, University of Padova, Padova, Italy.

Zoltan Geiger. University of Debrecen, Faculty of Medicine, Department of Internal Medicine, Division of Clinical Immunology, Debrecen, Hungary.

Peter Gregersen. The Robert S. Boas Center for Genomics and Human Genetics, The Feinstein Institute, Manhasset, New York.

Michael G. Hanna. Department of Neuromuscular Diseases, UCL Queen Square Institute of Neurology, and Centre for Rheumatology, UCL Division of Medicine, University College London, London, UK.

Janine A Lamb. Epidemiology and Public Health Group, School of Health Sciences, University of Manchester, Manchester, UK.

Vidya Limaye. Rheumatology Unit, Royal Adelaide Hospital and Discipline of Medicine, Adelaide University, Adelaide, Australia.

Ingrid Lundberg. Division of Rheumatology, Department of Medicine, Solna, Karolinska Institutet, Karolinska University Hospital, Stockholm, Sweden.

Pedro Machado. Department of Neuromuscular Diseases, UCL Queen Square Institute of Neurology, and Centre for Rheumatology, UCL Division of Medicine, University College London, London, UK.

Pernille Mathiesen. Paediatric Department, Slagelse Hospital and Paediatric Rheumatology Unit, Rigshospitalet, Copenhagen, Denmark.

Britta Maurer. Department of Rheumatology and Immunology, University Hospital, Bern, Switzerland.

Frederick W. Miller. Environmental Autoimmunity Group, National Institute of Environmental Health Sciences, NIH, Bethesda, Maryland.

Øyvind Molberg. Department of Rheumatology, Oslo University Hospital, Oslo, Norway.

Lauren Pachman. Ann & Robert H. Lurie Children's Hospital of Chicago, Northwestern University Feinberg School of Medicine, Chicago, Illinois.

Leonid Padyukov: Division of Rheumatology, Department of Medicine, Solna, Karolinska Institutet, Karolinska University Hospital, Stockholm, Sweden.

Timothy Radstake. Department of Rheumatology and Clinical Immunology, University Medical Center, Utrecht, the Netherlands.

Ann M Reed. Department of Pediatrics, Duke University, Durham, North Carolina.

Lisa G. Rider. Environmental Autoimmunity Group, National Institute of Environmental Health Sciences, NIH, Bethesda, Maryland.

Simon Rothwell: Centre for Genetics and Genomics Versus Arthritis, Centre for Musculoskeletal Research, Faculty of Biology, Medicine and Health, University of Manchester, Manchester, UK.

Adam Schiffenbauer. Environmental Autoimmunity Group, National Institute of Environmental Health Sciences, NIH, Bethesda, Maryland.

Albert Selva-O’Callaghan. Internal Medicine Department, Vall d'Hebron General Hospital, Universitat Autonoma de Barcelona, Barcelona, Spain.

Jiri Vencovksy. Institute of Rheumatology and Department of Rheumatology, First Medical Faculty, Charles University, Prague, Czech Republic.

Lucy R Wedderburn. NIHR Biomedical Research Centre at Great Ormond Street Hospital, and Arthritis Research UK Centre for Adolescent Rheumatology, UCL Great Ormond Street Institute of Child Health, University College London, London, UK.

Catherine Zhu. Baylor College of Medicine, Houston, Texas.

SUPPLEMENTARY APPENDIX II: Investigators from InterLymph Consortium contributing to the genetics studies of B-cell lymphomas

Sonja Berndt. Division of Cancer Epidemiology and Genetics, National Cancer Institute, Rockville, MD, USA.

Parveen Bhatti. School of Population and Public Health, University of British Columbia, Vancouver, BC, V6T 1Z3, Canada. Population Health Sciences, BC Cancer Research Institute, Vancouver, BC, V5Z 1L3, Canada.

Lauren Teras. Department of Population Science, American Cancer Society, Atlanta, GA, United States.

Alain Monnereau. Bordeaux Population Health Research Center, EPICENE, UMR 1219, Univ. Bordeaux, Inserm, Bordeaux, France; Registre des Hémopathies Malignes De La Gironde - Institut Bergonié, Bordeaux, France.

Roel Vermeulen. Institute for Risk Assessment Sciences, Division of Environmental Epidemiology and Julius Center for Health Sciences and Primary Care, University Medical Center Utrecht, Utrecht University, Utrecht, Netherlands.

Delphine Casabonne. Spanish Consortium for Research on Epidemiology and Public Health (CIBERESP), Madrid, Spain; Unit of Molecular and Genetic Epidemiology in Infections and Cancer (UNIC-Molecular), Cancer Epidemiology Research Programme, IDIBELL, Catalan Institute of Oncology, Becelona, Spain.

Alexandra Nieters. Institute for Immunodeficiency, Medical Centre and Faculty of Medicine, Albert-Ludwigs-University, Freiburg, Germany.

James McKay. International Agency for Research on Cancer, World Health Organization, Lyon, France.

Brenda Birmann. Channing Division of Network Medicine, Department of Medicine, Brigham and Women's Hospital and Harvard Medical School, Boston, MA, USA.

Pierluigi Cocco. Department of Medical Sciences and Public Health, University of Cagliari, Cagliari, Italy.

James Cerhan. Department of Quantitative Health Sciences, Mayo Clinic, Rochester, MN, USA.

Brian Link. Division of Hematology, Oncology and Bone Marrow Transplantation, University of Iowa, Iowa City, IA, USA.

Roger Milne. Cancer Council Victoria, East Melbourne, Victoria, Australia; Centre for Epidemiology and Biostatistics, Melbourne School of Population and Global Health, The University of Melbourne, Victoria, Australia; Precision Medicine, School of Clinical Sciences at Monash Health, Monash University, Clayton, Victoria, Australia.

Harindra Jayasekara. Cancer Epidemiology Division, Cancer Council Victoria, Melbourne, Victoria, Australia.Centre for Epidemiology and Biostatistics, Melbourne School of Population and Global Health, The University of Melbourne, Melbourne, Victoria, Australia. School of Public Health and Preventive Medicine, Faculty of Medicine, Nursing and Health Sciences, Monash University, Clayton, Victoria, Australia.

Vijai Joseph. Memorial Sloan-Kettering Cancer Center, New York, NY, USA.

Kenneth Offit. Memorial Sloan-Kettering Cancer Center, New York, NY, USA.

Nathaniel Rothman. Division of Cancer Epidemiology and Genetics, National Cancer Institute, NIH, DHHS, Rockville, MD, USA.

Claire Vajdic. Kirby Institute, UNSW Sydney, Sydney, Australia.

Alan Arslan. Departments of Obstetrics and Gynecology and Population Health, New York University Grossman School of Medicine, New York, NY, USA.

Mark Purdue. Division of Cancer Epidemiology and Genetics, National Cancer Institute, NIH, DHHS, Rockville, MD, USA.

Henrik Hjalgrim. Haematology, Danish Cancer Institute, Copenhagen, Denmark. Department of Haematology, Rigshospitalet, University of Copenhagen, Copenhagen, Denmark. Department of Clinical Medicine, University of Copenhagen, Copenhagen, Denmark.

Karin Ekstrom Smedby. Clinical epidemiology division, Department of Medicine, Solna, Karolinska Institutet, Stockholm, Sweden. Hematology Center, Karolinska University Hospital, Stockholm, Sweden.

Paige Bracci. Department of Epidemiology and Biostatistics, University of California, San Francisco, San Francisco, California, USA.

Eleanor Kane. Epidemiology and Cancer Statistics Group, Department of Health Sciences, University of York, York, UK.

Lesley Tinker. Division of Public Health Sciences, Women's Health Initiative Clinical Coordinating Center, Fred Hutchinson Cancer Center, Seattle, WA, USA.

Qing Lan. Division of Cancer Epidemiology and Genetics, National Cancer Institute, NIH, DHHS, Rockville, MD, USA.

Susan Slager. Department of Quantitative Health Sciences, Mayo Clinic, Rochester, Minnesota, USA. Division of Hematology, Mayo Clinic, Rochester, Minnesota, USA.

Immaculata De Vivo. Channing Division of Network Medicine, Department of Medicine, Brigham and Women's Hospital and Harvard Medical School, Boston, MA, USA; Department of Epidemiology, Harvard T.H. Chan School of Public Health, Boston, MA, USA.

Gilles Salles. Department of Medicine, Lymphoma Service, Memorial Sloan Kettering Cancer Center, New York, NY, USA. Department of Medicine, Weill Cornell Medical College, New York, NY, USA.

Nicola Camp. Huntsman Cancer Institute, Salt Lake City, UT 84108, USA; Department of Internal Medicine, University of Utah School of Medicine, Salt Lake City, UT 84108, USA.

SUPPLEMENTARY APPENDIX III: Funding information for contributing GWASs

The previous GWAS and ImmunoChip studies of IIM are supported by the Association Francaise Contre Les Myopathies (AFM); European Union Sixth Framework Programme (project AutoCure; LSH-018661); European Science Foundation (ESF) in the framework of the Research Networking Programme European Myositis Network (EUMYONET); European Reference Network for Rare Neuromuscular Diseases (ERN EURO-NMD); The Swedish Research Council and grants provided by the Stockholm County Council (ALF project), the Swedish Rheumatism Association, King Gustaf V 80 year Foundation; the intramural research program of the National Institute of Environmental Health Sciences (NIEHS), the National Institutes of Health (NIH) (Project ZIA ES101074) ; Myositis UK; Arthritis Research UK (awards 18474, 21593, 21552 and 20380); Medical Research Council (MR/N003322/1); The Cure JM Foundation; the Wellcome Trust (awards 085860 and 105610); the Henry Smith Charity UK; Action Medical UK; the Remission Charity; BMA Dorris Hillier Grant 2012 and the Bath Institute of Rheumatic Diseases; Prinses Beatrix Spierfonds (W.OR12-15); the Research Fund of Region Zeeland, Denmark; the Ministry of Health, Czech Republic (Project for Conceptual Development of Research Organization grant 00023728); National Institute for Health Research (NIHR) Biomedical Research Centres (BRC) at Great Ormond Street Hospital (GOSH), Manchester and University College London Hospitals. The contributions of the NIH author(s) were made as part of their official duties as NIH federal employees, are in compliance with agency policy requirements, and are considered Works of the United States Government. However, the findings and conclusions presented in this paper are those of the author(s) and do not necessarily reflect the views of the NIH or the U.S. Department of Health and Human Services. The InterLymph consortium is

supported by the Intramural Research Program of the Division of Cancer Epidemiology and Genetics, National Cancer Institute, National Institute of Health. These funding sources were not involved in any parts of the presented research.

REFERENCES

1. Ormond C, Ryan NM, Corvin A, Heron EA. Converting single nucleotide variants between genome builds: from cautionary tale to solution. *Brief Bioinform*. Sep 2 2021;22(5)doi:10.1093/bib/bbab069
2. *liftOver: Changing genomic coordinate systems with rtracklayer::liftOver*. R package version 1.24.0; 2023. https://www.bioconductor.org/help/workflows/liftOver/
3. *R: A language and environment for statistical computing*. R Foundation for Statistical Computing; 2021. URL https://www.R-project.org/
4. Murphy AE, Schilder BM, Skene NG. MungeSumstats: a Bioconductor package for the standardization and quality control of many GWAS summary statistics. *Bioinformatics*. Dec 7 2021;37(23):4593-4596. doi:10.1093/bioinformatics/btab665
5. *MungeSumstats: Standardise summary statistics from GWAS*. R package version 1.2.4; 2022. https://github.com/neurogenomics/MungeSumstats
