# Supplementary material for: Identification of genetic variants associated with idiopathic inflammatory myopathies via cross-trait analysis with B cell lymphomas
Source: HGG Adv. 2026 Jul 24;7(4):100658. doi: 10.1016/j.xhgg.2026.100658 (PMC13495596; doi:10.1016/j.xhgg.2026.100658)
Supplement: Supplementary file 2 [file mmc3.pdf]

HGG Advances

ARTICLE

Identification of genetic variants associated with idiopathic inflammatory myopathies via cross-trait analysis with B cell lymphomas

Weng Ian Che,[1] James N. Jarvis,[2] Anton Öberg Sysojev,[3] Catherine Zhu,[4] Karina Patasova,[5,6,7] International Lymphoma Epidemiology Consortium (InterLymph),[12] IMACS Genetics Group (MYOGEN),[12] Karin E. Smedby,[3,8] Ingrid E. Lundberg,[9,10] Helga Westerlind,[3] Janine A. Lamb,[11,13] and Marie Holmqvist[3,10,13,14,*]

Summary

The genetic architecture of idiopathic inflammatory myopathies (IIMs) remains incompletely defined. When increasing sample size is not feasible, cross-trait analysis of genetically correlated diseases offers an effective strategy for discovering risk loci. Using summary statistics of IIM and B cell lymphoma subtypes, we applied conditional false discovery rate (condFDR) and multi-trait analysis of genome-wide association studies (GWASs) (MTAG) to detect genetic associations with IIM risk. Single-nucleotide polymorphisms (SNPs) outside the human leukocyte antigen (HLA) region meeting significance thresholds (condFDR < 0.01 or $p < 5 \times 10^{-8}$ for MTAG) were clumped and subjected to both functional annotation in FUMA and Gene Ontology (GO) biological process enrichment analysis using clusterProfiler. We identified six previously unreported loci, including three associated with dermatomyositis (chr12:58674304T>C, chr13:110799415C>T, and chr17:38103285G>A (hg19)) and three associated with polymyositis (PM) (chr4:971496T>C, chr6:396321C>T, and chr6:32650631C>A). All non-HLA loci act as expression quantitative trait loci (eQTL) or localize within enhancer regions. Notably, these include *cis*-eQTLs for known IIM risk-associated genes (*GSDMB*, *DGKQ*, *SLC26A1*, and *IDUA*) as well as genes implicated in synaptic vesicle cycle (SVC) pathways, immune regulation (*IKZF3*, *ORMDL3*, *IRF4*, *DUSP22*, and *SPON2*), protein homeostasis (*ATP23* and *PSMD3*), lipid metabolism (*PGAP3*, *ORMDL3*, *STARD3*, and *DGKQ*), and myopathy (*COL4A1*). GO analysis revealed significance for SVC pathways in PM (FDR < 0.05). These findings advance our understanding of IIM pathogenesis from a genetic perspective and highlight candidate regulatory variants for further mechanistic investigation.

Introduction

Idiopathic inflammatory myopathies (IIMs) encompass a spectrum of rare rheumatic diseases characterized by diverse phenotypes, with most cases sharing muscle inflammation to varying extent as a common feature. Dermatomyositis (DM) and polymyositis (PM) are the two broad clinical subtypes traditionally defined based on the Bohan and Peter criteria, with PM being distinct from DM by the absence of skin manifestations. In recent years, the discovery of IIM-related autoantibodies linked to distinct clinical features and outcomes has led to the recognition of additional clinical subtypes. Many PM cases defined in previous studies could actually represent other IIM subtypes, including anti-synthetase syndrome (ASyS), immune-mediated necrotizing myopathy (IMNM), inclusion body myositis (IBM), and overlap myositis.[1]

While the pathogenesis of IIMs remains unclear, it has been suggested that IIMs may be triggered by environmental exposures in genetically susceptible individuals.[1] Genome-wide association studies (GWASs) and fine-mapping studies performed with ImmunoChip in patients with IIMs published in the last decade have opened a door to understanding the genetic contribution to disease development, identifying alleles in the human leukocyte antigen (HLA) 8.1 ancestral haplotype as the strongest genetic risk factors.[2,3] However, a gap in heritability exists, evidenced by the difference between the family-based heritability (22%) and ImmunoChip single-nucleotide polymorphism (SNP)-based heritability (5.5% for DM and 8.3% for PM).[4,5] This discrepancy suggests that additional genetic variants associated with IIM risk remain unidentified. Discovering mild to moderate genetic associations with IIM risk may require larger sample sizes than

[1]Department of Public Health and Medicinal Administration, Faculty of Health Sciences, University of Macau, Macau, Macau SAR, China; [2]Department of Pediatrics and Center for Indigenous Health, University of Washington School of Medicine, Seattle, WA, USA; [3]Clinical Epidemiology Division, Department of Medicine, Solna, Karolinska Institutet, Stockholm, Sweden; [4]Institute for Clinical and Translational Research, Baylor College of Medicine, Houston, TX, USA; [5]Center for Molecular Medicine, Department of Medicine, Solna, Karolinska Institutet, Stockholm, Sweden; [6]Translational and Clinical Research Institute, Newcastle University, Newcastle upon Tyne, UK; [7]Population Health Sciences Institute, Newcastle University, Newcastle upon Tyne, UK; [8]Hematology Center, Karolinska University Hospital, Stockholm, Sweden; [9]Division of Rheumatology, Department of Medicine, Solna, Karolinska Institutet, Stockholm, Sweden; [10]ME Gastro, Derm and Rheuma, Theme Inflammation and Aging, Karolinska University Hospital, Stockholm, Sweden; [11]Epidemiology and Public Health Group, School of Health Sciences, The University of Manchester, Manchester, UK
[12]Further details can be found in the supplemental information
[13]These authors contributed equally
[14]Lead contact
*Correspondence: marie.holmqvist@ki.se
https://doi.org/10.1016/j.xhgg.2026.100658.
© 2026 The Author(s). Published by Elsevier Inc. on behalf of American Society of Human Genetics.
This is an open access article under the CC BY license (http://creativecommons.org/licenses/by/4.0/).

previous genetic studies, which is challenging for such a rare disease. Nevertheless, researchers have successfully identified numerous non-HLA genetic variants linked to the risk of IIMs through meta-analysis of already genotyped SNPs from related rheumatic diseases, genome-wide imputation, and cross-trait analysis of GWAS summary statistics with immune-related diseases.[6–9]

B cell lymphomas, malignancies arising from different stages of B lymphocyte maturation, exhibit shared immunogenetic characteristics with IIMs and have been consistently linked to DM and PM.[1,10–12] While significant associations with diffuse large B cell lymphoma (DLBCL) and follicular lymphoma (FL) have been reported in patients with IIMs,[13] evidence for elevated risks of chronic lymphocytic leukemia (CLL) and marginal zone lymphoma (MZL) remains limited.[14] Nevertheless, in our previous work demonstrating shared genetic susceptibility between common IIM and B cell lymphoma subtypes, strong pleiotropic enrichments of SNPs with low *p* values for DM and PM when conditioned on CLL SNPs with *p* values of <0.001 were observed.[12] Given the observed relationship between IIMs and B cell lymphomas, we cross-analyzed summary statistics from published genetic studies of IIM and four common B cell lymphoma subtypes, including DLBCL, FL, CLL, and MZL, to identify genetic variants associated with the risk of IIMs.

Material and methods

Data sources

Summary statistics of genome-wide SNP associations with DM and PM were obtained from the Genetics Scientific Interest Group (MYOGEN) of the International Myositis Assessment & Clinical Studies Group (IMACS). These summary-level data were derived from meta-analyses of prior GWASs[2] and ImmunoChip studies,[3] separately imputed to the TOPMed reference panel (version R2).[9] Details of the imputation and quality control procedures have been described previously.[9] In brief, variants were retained if they had imputation quality ≥0.6, minor allele frequency ≥0.005, and passed Hardy-Weinberg equilibrium testing in controls. No patients with IBM were included in the PM group of the GWAS and ImmunoChip studies.[2,3] The DM and PM cohort comprised 1,131 and 1,094 individuals, respectively, and 11,697 individuals in the control group. Summary statistics for SNPs linked to the risks of DLBCL, FL, CLL, and MZL resulting from previous GWASs were obtained from the International Lymphoma Epidemiology Consortium (InterLymph).[15–18] The CLL cohort included approximately 30% of individuals with small lymphocytic lymphoma, representing the same disease entity without peripheral blood lymphocytosis.[19] Among B cell lymphoma subtypes, the group numbers were 3,587 (DLBCL), 2,847 (FL), 3,100 (CLL), and 825 (MZL), with control sample sizes ranging from approximately 6,000 to 8,000. All genetic data were obtained from individuals of European ancestry. Potential population stratification was accounted for in the original GWAS analyses.[2,3,9,15–18] After additional quality control and standardization, summary statistics in the hg19 genome build for DM and PM from the meta-analysis study were paired to each B cell lymphoma subtype, resulting in a total of eight disease pairs (four per IIM subtype; see supplemental methods and Table S1).

Statistical methods

To identify genetic variants associated with DM and PM risk, we applied conditional false discovery rate (condFDR)[20] and multi-trait analysis of GWASs (MTAG),[21] which leverage genetic correlation across traits to increase statistical power under distinct modeling frameworks. These approaches enable the detection of SNPs associated with IIM risk that may not have reached genome-wide significance (*p* value $<5 \times 10^{-8}$) in single-trait analyses due to limited power. Analyses were restricted to SNPs shared between each disease pair and aligned to the reference panel (Table S2).

CondFDR is a model-free method that recalibrates nominal *p* values for SNP associations with a primary phenotype by leveraging genetic pleiotropy with a secondary phenotype.[20] We computed the condFDR of SNPs of a given IIM subtype based on the conditional empirical cumulative distribution function (cdf) of the *p* values of the corresponding SNPs of a given B cell lymphoma subtype. To mitigate false-positive findings, nominal *p* values of SNPs were adjusted using intergenic inflation control. SNPs within the HLA and chromosome 8p23.1 regions were excluded from fitting the conditional empirical cdf but retained for discovery analyses. A conservative significance threshold of condFDR <0.01 was applied.

MTAG exploits correlations in GWAS effect estimates arising from shared genetic architecture or correlated estimation errors across traits to improve statistical efficiency.[21] We meta-analyzed summary statistics for DM and PM separately with those of the four B cell lymphoma subtypes, using a genome-wide significance threshold of $p < 5 \times 10^{-8}$. Because MTAG assumes a common variance-covariance structure of SNP effect sizes across traits, violation of this assumption may inflate type I error. This potential bias was assessed by computing the maximum FDR (maxFDR) under a multivariate spike-and-slab model.[21] Given the substantial computational burden for more than two traits, we calculated pairwise maxFDR for each DM and PM disease pair and retained the maximum value.

Among SNPs reaching statistical significance in either analysis, we identified the lead SNPs through clumping ($r^2 < 0.1$ in linkage disequilibrium [LD] windows of 10,000 kb and merged loci closer than 250 kb). To elucidate the functional relevance of the previously unreported non-HLA loci identified in our study, FUMA SNP2GENE (version 2.0.0) was used for positional mapping of lead SNPs and their high-LD proxies ($r^2 > 0.8$). For these identified variants, we further performed *cis*-acting expression quantitative trait locus (*cis*-eQTL) analysis to identify variants regulating gene expression within a 1-Mb window and used Hi-C chromatin interaction mapping to detect long-range spatial contacts between genomic regions.[22] The direction of eQTL effect was available only for variants included in our dataset. Parameter settings are described in Table S3. In brief, mappings considered only protein-coding genes, with significant eQTL association (FDR < 0.05) and chromatin interactions (FDR $< 1 \times 10^{-6}$, a recommended stringent threshold to ensure most confident Hi-C contacts[23]) retained. Chromatin interactions were further filtered to include only interactions between enhancers (regions that overlap with the tested SNP) and promoter regions of genes. Roadmap Epigenomics Projects for 111 epigenomes was used for enhancer and promoter annotations.[24] All eQTL and Hi-C datasets used are of high quality and come from tissue or cell types relevant to IIMs.[22]

Gene Ontology (GO) enrichment analysis (biological process only) was performed separately for functionally mapped DM

Table 1. Loci associated with dermatomyositis and polymyositis risk identified by conditioning on four common B cell lymphoma subtypes

Disease	Conditioning disease	SNP ID	chr:bp in hg19	Function	Reference allele	Effect allele	Z score	pvalue[a]	CondFDR	Nearest protein-coding genes[b]
Dermatomyositis	FL	rs117912338	12:58674304	intergenic	T	C	4.5289	1.78×10^{-5}	0.0058	–
	DLBCL, CLL, MZL	rs2084040	13:110799415	intergenic	C	T	5.3744	3.51×10^{-7}	0.0020	*COL4A1*
	MZL	rs8080734	17:38103285	intergenic	G	A	−4.7510	6.74×10^{-6}	0.0094	*ORMDL3, LRRC3C, GSDMA*
Polymyositis	FL, CLL	rs116747058	4:971496	intergenic	T	C	5.0740	3.51×10^{-6}	0.0045	*DGKQ, SLC26A1, IDUA, GAK*
	CLL	rs12203592	6:396321	intronic	C	T	5.1260	2.78×10^{-6}	0.0002	***IRF4***

SNP, single-nucleotide polymorphism; DLBCL, diffuse large B cell lymphoma; FL, follicular lymphoma; CLL, chronic lymphocytic leukemia; MZL, marginal zone lymphoma; chr:bp, chromosome:basepairs; CondFDR, conditional false discovery rate.

[a]The *p* values were adjusted using intergenic inflation control.

[b]The lead SNPs and non-GWAS-tagged candidate SNPs in linkage disequilibrium ($r^2 > 0.8$) with the lead SNPs were mapped in this positional mapping. The gene containing an intronic lead SNP is highlighted in bold.

and PM risk-associated genes using clusterProfiler and the org.Hs.eg.db annotation packages.[25,26] Significance was defined as an FDR of <0.05. All GO terms, regardless of statistical significance, were then simplified by semantic similarity-based clustering (threshold = 0.7) using rrvgo.[27] Gene set enrichment analyses based on Kyoto Encyclopedia of Genes and Genomes (KEGG), Reactome, and WikiPathways were performed using FUMA GENE2FUNC (version 2.0.0), with significance defined as a Benjamini-Hochberg adjusted *p* value of <0.05.[22]

This study was approved by the Swedish Ethical Review Authority (2020-04529 and 2023-01343-02).

Results

In the condFDR analyses, five loci each were identified for DM and PM with condFDR <0.01 when conditioning on the four B cell lymphoma subtypes (Tables 1, S4, S5, and S6). Of these, three (DM) and two (PM) represent candidate loci not previously reported at genome-wide significance. All lead SNPs reside in non-coding regions, predominantly intergenic. Except for SNP chr12:58674304T>C (DM), all lead variants had *p* values below the suggestive association threshold (1×10^{-5}) in the IIM meta-analysis study.[9] No identified loci were shared between DM and PM. Notably, variant chr13:110799415C>T, associated with DM risk, was identified across multiple conditioning analyses (DLBCL, CLL, and MZL), whereas the PM risk-associated variant chr4:971496T>C was consistently detected when conditioning on FL and CLL.

In the MTAG analyses, all genome-wide significant SNPs associated with DM risk were located within the HLA region and corresponded to previously reported loci. For PM, a previously unreported locus reaching genome-wide significance (lead SNP chr6:32650631C>A, intergenic; *p* value = 1.44×10^{-8}) within the HLA class II region was identified, with a maxFDR of 0.0003.

Functional annotation for DM-associated variants

All identified DM risk-associated lead SNPs function as *cis*-eQTLs in IIM-relevant tissues/cell types, with the direction of effect specified (Figure 1 and Table S7). The variant chr12:58674304T>C downregulates *ATP23* (>320 kb upstream; MIM: 619760), while an LD proxy of chr13:110799415C>T regulates the nearby gene *COL4A1* (MIM:120130). The variant chr17:38103285G>A shows complex regulatory effects: it upregulates *CDK12* (MIM: 615514) and *MIEN1* (MIM: 611802); downregulates *KRT10-AS1*, *PSMD3* (MIM: 617676), *SMARCE1* (MIM: 603111), and *STARD3* (MIM: 607048); and bidirectionally regulates *PGAP3* (MIM: 611801), *GSDMA* (MIM: 611218), *GSDMB* (MIM: 611221), *IKZF3* (MIM: 606221), *MED24* (MIM: 607000), and *ORMDL3* (MIM: 610075). Beyond blood, regulatory effects were also observed in fibroblasts, gastrointestinal tissues, and adipose tissue, affecting genes such as *GSDMA* (MIM: 611218), *GSDMB* (MIM: 611221), *MED24* (MIM: 607000), and *ORMDL3* (MIM: 610075). Hi-C chromatin interaction mapping further linked

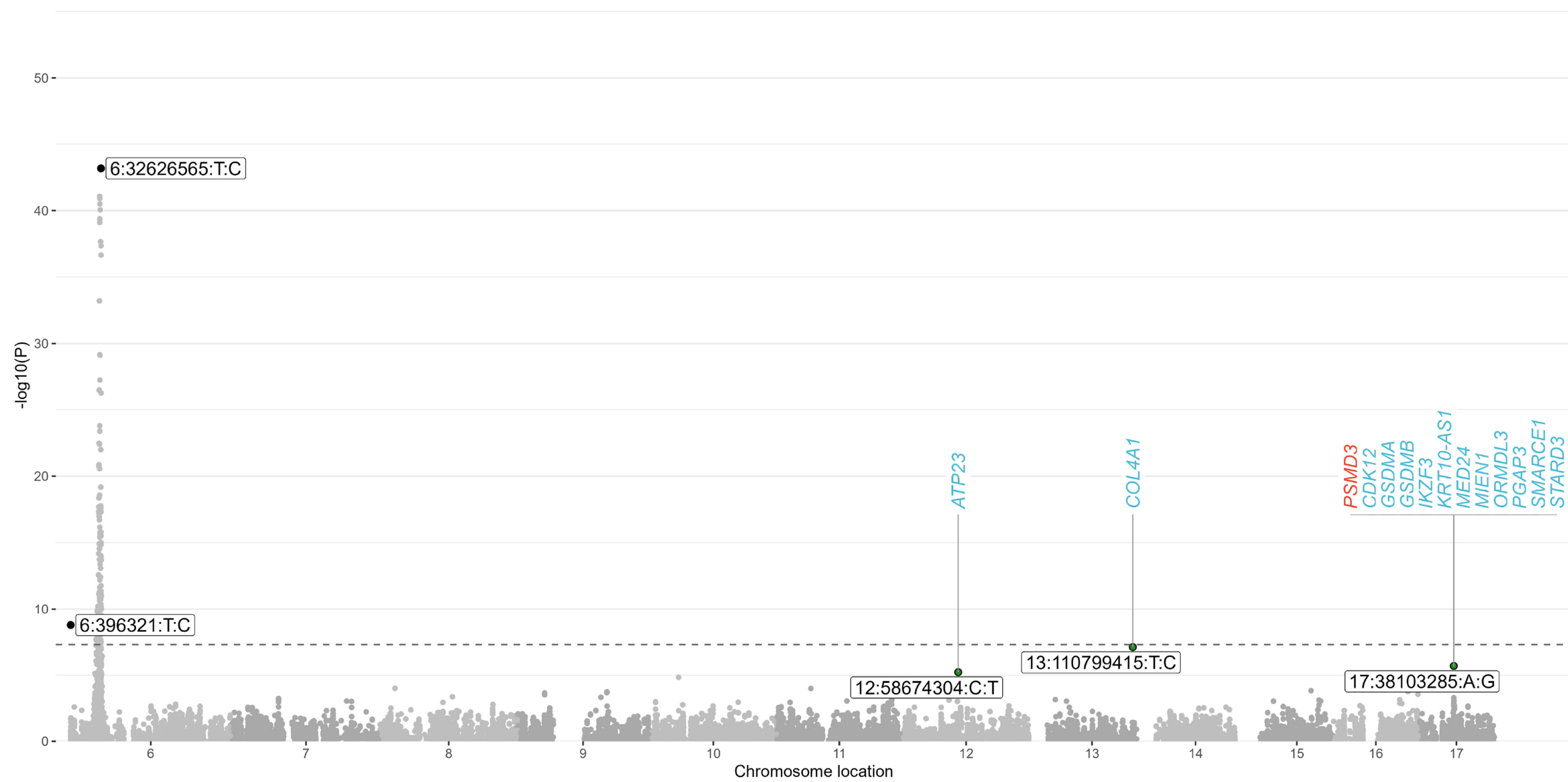

Figure 1. Manhattan plot of dermatomyositis risk-associated loci identified in the conditional false discovery rate (condFDR) analysis (condFDR < 0.01), with functionally mapped genes annotated for previously unreported loci
Genes supported by eQTL evidence only are shown in blue, while those supported by both eQTL and Hi-C evidence are shown in red. The *x* axis of the plot is truncated to focus on chromosomes with statistically significant signals. eQTL, expression quantitative trait locus.

chr17:38103285G>A to *PSMD3* (MIM: 617676) in brain tissue (Figure 1 and Table S8).

The 14 genes mapped via eQTL associations or chromatin interactions for the three DM risk loci and their LD proxies were annotated to 253 GO biological processes (Table S9). Many of these processes were related to lipid metabolic and biosynthetic processes and immune regulation, although none showed significant enrichment. No significant results were identified in KEGG, Reactome, or WikiPathways enrichment analyses.

Functional annotation for PM-associated variants

The two PM risk-associated lead SNPs function as *cis*-eQTLs in blood (Figure 2 and Table S10). The variant chr4:971496T>C was associated with regulation of multiple genes, including upregulation of *CTBP1* (MIM: 602618), *IDUA* (MIM: 252800), and *SPON2* (MIM: 605918); downregulation of *PCGF3* (MIM: 617543), *RNF212* (MIM: 612041), and *UVSSA* (MIM: 614632); and bidirectional regulation of *DGKQ* (MIM: 601207). The variant chr6:396321C>T, previously linked to IIM overall, DM, and juvenile DM,[9] was here associated with PM and shows *cis*-eQTL effects on *EXOC2* (MIM: 615329) and *MATN2* (MIM: 602108) in addition to *IRF4* (MIM: 601900). Chromatin interaction analyses further linked this variant to *DUSP22* (MIM: 616778) in spleen and lymphoblastoid cells (Figure 2 and Table S11).

Eleven genes identified through functional mapping for the two PM risk loci were annotated to 394 GO biological processes, with four synaptic vesicle cycle (SVC) processes significantly enriched (gene set: *GAK* [MIM: 602052], *DGKQ* [MIM: 601207], and *CTBP1* [MIM: 602618]; fold enrichment: 57.3; adjusted p value $= 0.003$) (Table S12). Although they lack statistical significance, additional biological processes related to lipid metabolism and biosynthesis, and immune regulation, were also annotated. No significant enrichment was observed in KEGG, Reactome, or WikiPathways.

Discussion

In this study, we improved statistical efficiency in detecting genetic associations with IIM risk by using condFDR and MTAG, leveraging genetic correlation with B cell lymphomas. A major challenge in IIM genetics is the limited cohort size for identifying common variants with moderate effect sizes. Although prior epidemiological studies found no statistically significant familial associations between IIMs and B cell lymphomas (standardized incidence ratio/adjusted odds ratio ranging from 1.2 to 2.0),[28,29] we previously demonstrated locus-specific genetic correlations, primarily in the HLA region and several non-HLA regions.[12] Consistent with these findings, we identified a previously unreported HLA class II locus associated with PM at genome-wide significance (chr6:32650631C>A); however, the corresponding HLA allele and amino acid could not be determined due to the lack of HLA imputation data. The *cis*-eQTL chr6:396321C>T identified for PM, which has also reached genome-wide significance for DM and juvenile DM,[9] was previously shown to be jointly associated

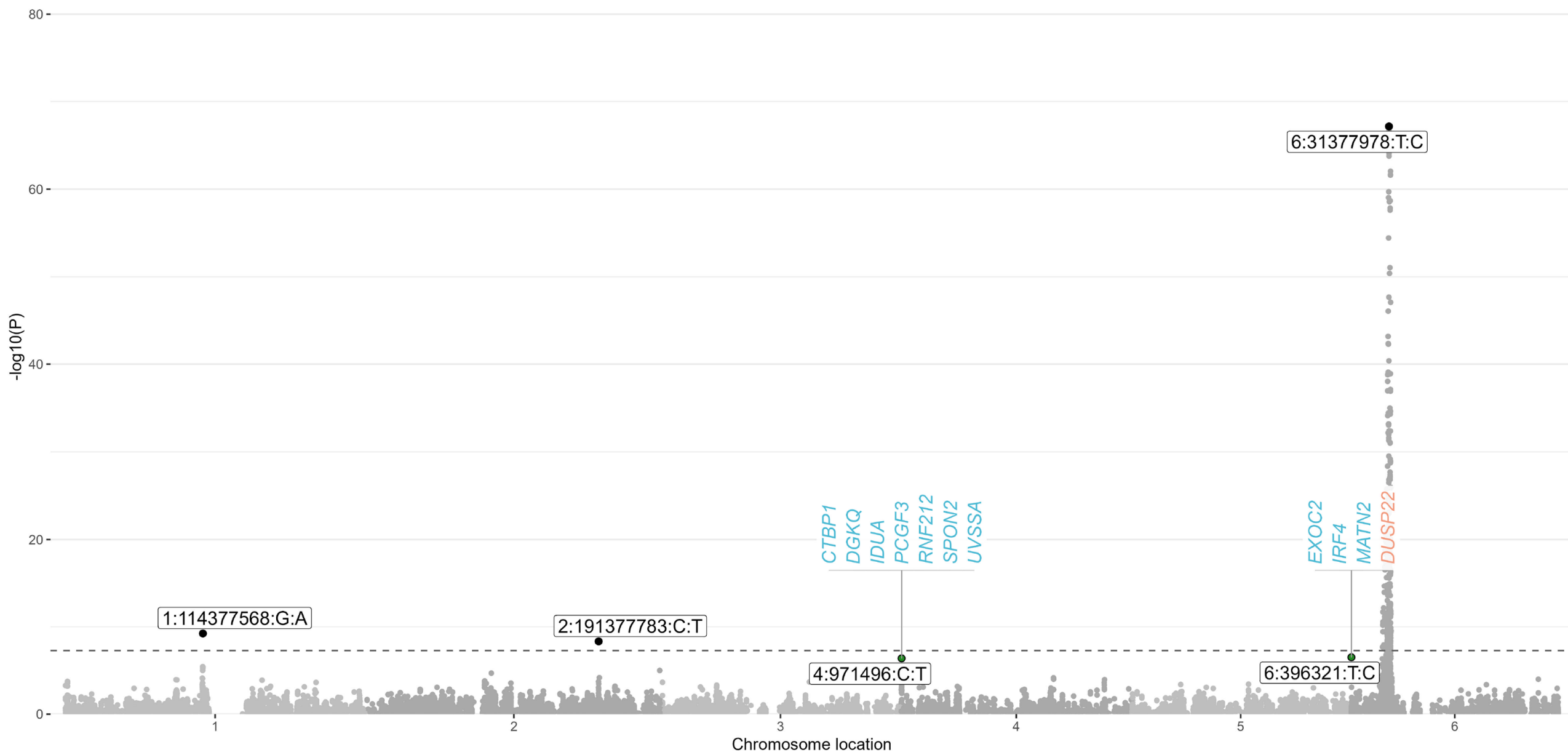

Figure 2. Manhattan plot of polymyositis risk-associated loci identified in the condFDR analysis (condFDR < 0.01), with functionally mapped genes annotated for previously unreported loci
Genes supported by eQTL evidence only are shown in blue, and those supported by Hi-C evidence only in coral. The *x* axis of the plot is truncated to focus on chromosomes with statistically significant signals. eQTL, expression quantitative trait locus.

with CLL for both DM and PM.[12] Earlier ImmunoChip-based analyses also suggested genetic correlations at regions chr1:205917549–208162951 (DM and DLBCL) and chr9:138995792–140097759 (PM and DLBCL). However, no SNP associations were identified within these regions in the present study. Nevertheless, genetic correlations between IIMs and B cell lymphomas may have been underestimated in previous analyses, as methods for estimating locus-specific genetic correlation generally have limited statistical power for binary traits.[30]

We replicated several previously reported non-HLA genetic associations for DM, PM, and IIM, including *GSDMB* (MIM: 611221), *DGKQ* (MIM: 601207), *SLC26A1* (MIM: 610130), and *IDUA* (MIM: 252800).[3,6,7] The *GSDMB* (MIM: 611221) eQTL variant chr17:38066267G>T, previously reported with suggestive significance for DM,[3] is in moderate LD ($r^2 = 0.5$) with our identified *GSDMB* (MIM: 611221) eQTL variant chr17:38103285G>A in European populations. For *DGKQ* (MIM: 601207), several eQTLs have been previously reported for IIMs.[3,6,7] Moreover, an intronic *SLC26A1* (MIM: 610130)/*IDUA* (MIM: 252800) variant has been linked to PM risk without a known functional role.[3] Here, we identified a previously unreported independent variant (chr4:971496T>C) acting as a *cis*-eQTL for *DGKQ* (MIM: 601207) and *IDUA* (MIM: 252800) in immune-related cells. These regulatory polymorphisms further support the involvement of these genes in IIM pathogenesis.

The PM risk-associated variant chr4:971496T>C was linked to genes involved in the SVC, including *DGKQ* (MIM: 601207), *CTBP1* (MIM: 602618), and *GAK* (MIM: 602052). Dysfunction of this pathway has been implicated in the accumulation of neurodegenerative proteins such as amyloid-beta and tau, pathological hallmarks shared between IBM skeletal muscles and Alzheimer disease brain tissue.[1,31] However, SVC-related mechanisms have not previously been implicated in PM or other non-DM IIM subtypes, warranting further investigation into their potential role in PM pathogenesis.

Another intriguing finding was the identification of variant mapping to genes involved in biological processes implicated in IIM pathogenesis. Several DM- and PM-associated genes participate in immune-related activities, including T cell and B cell regulation (DM: *IKZF3* [MIM: 606221] and *ORMDL3* [MIM: 610075]; PM: *IRF4* [MIM: 601900] and *DUSP22* [MIM: 616778]) and cytokine production (PM: *SPON2* [MIM: 605918]), key components of IIM pathophysiology, and therapeutic targets.[32] Additionally, DM-associated genes *ATP23* (MIM: 619760) and *PSMD3* (MIM: 617676) are linked to protein homeostasis. *PSMD3* (MIM: 617676), supported by both eQTL and chromatin interaction evidence, encodes a 26S proteasome regulatory subunit involved in the ubiquitin-proteasome pathway. These findings align with prior reports of dysregulated proteosome- and ubiquitination-related protein expression in peripheral blood of patients with DM.[33] Furthermore, a protein-protein interaction and pathway analysis of IIM-related autoantigens and proteins encoded by IIM risk-associated loci revealed direct interactions among ubiquitin proteasome pathway proteins, including PSMD3.[34]

We also identified DM risk-associated genes *PGAP3* (MIM: 611801), *ORMDL3* (MIM: 610075), and *STARD3* (MIM: 607048), and the PM risk-associated gene *DGKQ*

(MIM: 601207), related to lipid metabolism and biosynthesis, supporting accumulating evidence of lipid dysregulation across IIM subtypes.[35–39]

Notably, *COL4A1* (MIM: 120130), encoding the collagen type IV alpha 1 chain, a key structural component of the basement membrane, was identified as a DM risk-associated gene. Pathogenic variants in *COL4A1* (MIM: 120130) cause a multi-organ disorder characterized by hereditary angiopathy, nephropathy, aneurysms, and muscle cramps.[40] Consistently, *Col4a1* mutant mouse models exhibit progressive skeletal muscle myopathy, potentially due to vascular defects.[41,42] These findings suggest a potential link between DM and COL4A1-related myopathy, which requires further investigation.

The previously unreported non-HLA loci identified via condFDR were not replicated in the MTAG analyses, likely reflecting lower statistical power gain with MTAG in this context. MTAG performance depends on the genetic covariance across traits,[43] and as shared genetic susceptibility between IIMs and B cell lymphomas is primarily found within the HLA region,[12] gains for non-HLA locus discovery were limited.

This study has several limitations. First, independent replication was not feasible due to the rarity and heterogeneity of IIMs, and additional functional validation (e.g., fine-mapping and colocalization) was constrained by the limited statistical power of the original DM and PM meta-analyses. Future validation will require larger cohorts through international collaboration. Second, PM in the MYOGEN dataset was defined using the Bohan and Peter criteria, potentially including non-DM IIM subtypes such as IMNM and ASyS. While IBM and anti-Jo1-positive cases were excluded where applicable,[2,3] the identified PM risk-associated variants may reflect combined effects across PM, IMNM, ASyS, and overlap myositis. Finally, as the analyses were restricted to individuals of European ancestry, our findings may not generalize to other populations.

Despite these limitations, the application of power-enhancing analytical approaches combined with functional annotation enabled the identification of five previously unreported regulatory variants (three for DM and two for PM). These variants affect genes involved in SVC pathways, immune regulation, protein homeostasis, lipid metabolism and biosynthesis, and myopathy. Collectively, these findings advance the understanding of the genetic architecture underlying IIM pathogenesis and are useful for guiding future genetic studies.

Data and code availability

Due to data-use agreements with the International Myositis Assessment & Clinical Studies Group and the International Lymphoma Epidemiology Consortium, the summary statistics used in this study are not publicly available and can only be accessed with approval from the respective consortia. This study did not generate any new code.

Acknowledgments

This work was supported by the Swedish Research Council, the Swedish Cancer Foundation, the UM Macao Fellow scheme of the UM Talent Program of the University of Macau (file no. UMTP2023-MF02), and R01 AR078785 from the National Institutes of Health/National Institute of Arthritis and Musculoskeletal and Skin Diseases (J.N.J.). We would like to thank Lina Marcela Diaz-Gallo for outstanding advice on study design. We also want to acknowledge IMACS, the MYOGEN members, the LM GWAS Coordinating Committee, and the InterLymph Data Coordinating Committee for their indispensable support in data application and transfer and for critical review of the manuscript. Special thanks are extended to Arial Harrison, Dakai Zhu, and Dennis Robinson for assisting the data transfer. The contributing members of the IMACS MYOGEN and InterLymph Consortium are listed in the supplemental appendices.

Author contributions

All authors conceived and designed the work; W.I.C. curated and standardized the GWAS summary statistics, performed all analyses, interpreted the findings, and drafted the manuscript; and all authors contributed to revising the manuscript critically and intellectual content.

Declaration of interests

I.E.L. and M.H. have received grants from Swedish Research Council.

Supplemental information

Supplemental information can be found online at https://doi.org/10.1016/j.xhgg.2026.100658.

Web resources

OMIM, https://www.omim.org/

Received: January 30, 2026
Accepted: July 22, 2026

References

1. Lundberg, I.E., Fujimoto, M., Vencovsky, J., Aggarwal, R., Holmqvist, M., Christopher-Stine, L., Mammen, A.L., and Miller, F.W. (2021). Idiopathic inflammatory myopathies. Nat. Rev. Dis. Primers *7*, 86. https://doi.org/10.1038/s41572-021-00321-x.
2. Miller, F.W., Chen, W., O'Hanlon, T.P., Cooper, R.G., Vencovsky, J., Rider, L.G., Danko, K., Wedderburn, L.R., Lundberg, I.E., Pachman, L.M., et al. (2015). Genome-wide association study identifies HLA 8.1 ancestral haplotype alleles as major genetic risk factors for myositis phenotypes. Genes Immun. *16*, 470–480. https://doi.org/10.1038/gene.2015.28.
3. Rothwell, S., Cooper, R.G., Lundberg, I.E., Miller, F.W., Gregersen, P.K., Bowes, J., Vencovsky, J., Danko, K., Limaye, V., Selva-O'Callaghan, A., et al. (2016). Dense genotyping of immune-related loci in idiopathic inflammatory myopathies confirms HLA alleles as the strongest genetic risk factor and suggests different genetic background for major clinical

subgroups. Ann. Rheum. Dis. *75*, 1558–1566. https://doi.org/10.1136/annrheumdis-2015-208119.
4. Rothwell, S., Lamb, J.A., and Chinoy, H. (2016). New developments in genetics of myositis. Curr. Opin. Rheumatol. *28*, 651–656. https://doi.org/10.1097/BOR.0000000000000328.
5. Che, W.I., Westerlind, H., Lundberg, I.E., Hellgren, K., Kuja-Halkola, R., and Holmqvist, M. (2021). Familial aggregation and heritability: a nationwide family-based study of idiopathic inflammatory myopathies. Ann. Rheum. Dis. *80*, 1461–1466. https://doi.org/10.1136/annrheumdis-2021-219914.
6. Acosta-Herrera, M., Kerick, M., González-Serna, D., Myositis Genetics Consortium, Scleroderma Genetics Consortium, Wijmenga, C., Franke, A., Gregersen, P.K., Padyukov, L., Worthington, J., et al. (2019). Genome-wide meta-analysis reveals shared new loci in systemic seropositive rheumatic diseases. Ann. Rheum. Dis. *78*, 311–319. https://doi.org/10.1136/annrheumdis-2018-214127.
7. Rothwell, S., Amos, C.I., Miller, F.W., Rider, L.G., Lundberg, I.E., Gregersen, P.K., Vencovsky, J., McHugh, N., Limaye, V., Selva-O'Callaghan, A., et al. (2023). Identification of Novel Associations and Localization of Signals in Idiopathic Inflammatory Myopathies Using Genome-Wide Imputation. Arthritis Rheumatol. *75*, 1021–1027. https://doi.org/10.1002/art.42434.
8. Reales, G., Amos, C.I., Benveniste, O., Chinoy, H., De Bleecker, J., De Paepe, B., Doria, A., Gregersen, P.K., Lamb, J.A., Limaye, V., et al. (2024). Discovery of new myositis genetic associations through leveraging other immune-mediated diseases. HGG Adv. *5*, 100336. https://doi.org/10.1016/j.xhgg.2024.100336.
9. Zhu, C., Han, Y., Byun, J., Xiao, X., Rothwell, S., Miller, F.W., Lundberg, I.E., Gregersen, P.K., Vencovsky, J., Shaw, V.R., et al. (2025). Genetic Architecture of Idiopathic Inflammatory Myopathies From Meta-Analyses. Arthritis Rheumatol. *77*, 750–764. https://doi.org/10.1002/art.43088.
10. Smedby, K.E., and Ponzoni, M. (2017). The aetiology of B-cell lymphoid malignancies with a focus on chronic inflammation and infections. J. Intern. Med. *282*, 360–370. https://doi.org/10.1111/joim.12684.
11. Dani, L., Ian Che, W., Lundberg, I.E., Hellgren, K., and Holmqvist, M. (2021). Overall and site-specific cancer before and after diagnosis of idiopathic inflammatory myopathies: A nationwide study 2002-2016. Semin. Arthritis Rheum. *51*, 331–337. https://doi.org/10.1016/j.semarthrit.2020.12.009.
12. Che, W.I., Öberg Sysojev, A., Zhu, C., Patasova, K., International Lymphoma Epidemiology Consortium InterLymph, IMACS Myositis Genetics Scientific Interest Group MYOGEN, Smedby, K.E., Lundberg, I.E., Westerlind, H., and Holmqvist, M. (2025). Shared genetic susceptibility between idiopathic inflammatory myopathies and common B cell lymphoma subtypes found primarily in the human leucocyte antigen region. RMD Open *11*, e006035. https://doi.org/10.1136/rmdopen-2025-006035.
13. Hemminki, K., Huang, W., Sundquist, J., Sundquist, K., and Ji, J. (2020). Autoimmune diseases and hematological malignancies: Exploring the underlying mechanisms from epidemiological evidence. Semin. Cancer Biol. *64*, 114–121. https://doi.org/10.1016/j.semcancer.2019.06.005.
14. Anderson, L.A., Gadalla, S., Morton, L.M., Landgren, O., Pfeiffer, R., Warren, J.L., Berndt, S.I., Ricker, W., Parsons, R., and Engels, E.A. (2009). Population-based study of autoimmune conditions and the risk of specific lymphoid malignancies. Int. J. Cancer *125*, 398–405. https://doi.org/10.1002/ijc.24287.
15. Cerhan, J.R., Berndt, S.I., Vijai, J., Ghesquières, H., McKay, J., Wang, S.S., Wang, Z., Yeager, M., Conde, L., de Bakker, P.I.W., et al. (2014). Genome-wide association study identifies multiple susceptibility loci for diffuse large B cell lymphoma. Nat. Genet. *46*, 1233–1238. https://doi.org/10.1038/ng.3105.
16. Skibola, C.F., Berndt, S.I., Vijai, J., Conde, L., Wang, Z., Yeager, M., de Bakker, P.I.W., Birmann, B.M., Vajdic, C.M., Foo, J.N., et al. (2014). Genome-wide association study identifies five susceptibility loci for follicular lymphoma outside the HLA region. Am. J. Hum. Genet. *95*, 462–471. https://doi.org/10.1016/j.ajhg.2014.09.004.
17. Vijai, J., Wang, Z., Berndt, S.I., Skibola, C.F., Slager, S.L., de Sanjose, S., Melbye, M., Glimelius, B., Bracci, P.M., Conde, L., et al. (2015). A genome-wide association study of marginal zone lymphoma shows association to the HLA region. Nat. Commun. *6*, 5751. https://doi.org/10.1038/ncomms6751.
18. Berndt, S.I., Camp, N.J., Skibola, C.F., Vijai, J., Wang, Z., Gu, J., Nieters, A., Kelly, R.S., Smedby, K.E., Monnereau, A., et al. (2016). Meta-analysis of genome-wide association studies discovers multiple loci for chronic lymphocytic leukemia. Nat. Commun. *7*, 10933. https://doi.org/10.1038/ncomms10933.
19. Alaggio, R., Amador, C., Anagnostopoulos, I., Attygalle, A.D., Araujo, I.B.d.O., Berti, E., Bhagat, G., Borges, A.M., Boyer, D., Calaminici, M., et al. (2022). The 5th edition of the World Health Organization Classification of Haematolymphoid Tumours: Lymphoid Neoplasms. Leukemia *36*, 1720–1748. https://doi.org/10.1038/s41375-022-01620-2.
20. Andreassen, O.A., Thompson, W.K., Schork, A.J., Ripke, S., Mattingsdal, M., Kelsoe, J.R., Kendler, K.S., O'Donovan, M.C., Rujescu, D., Werge, T., et al. (2013). Improved detection of common variants associated with schizophrenia and bipolar disorder using pleiotropy-informed conditional false discovery rate. PLoS Genet. *9*, e1003455. https://doi.org/10.1371/journal.pgen.1003455.
21. Turley, P., Walters, R.K., Maghzian, O., Okbay, A., Lee, J.J., Fontana, M.A., Nguyen-Viet, T.A., Wedow, R., Zacher, M., Furlotte, N.A., et al. (2018). Multi-trait analysis of genome-wide association summary statistics using MTAG. Nat. Genet. *50*, 229–237. https://doi.org/10.1038/s41588-017-0009-4.
22. Watanabe, K., Taskesen, E., van Bochoven, A., and Posthuma, D. (2017). Functional mapping and annotation of genetic associations with FUMA. Nat. Commun. *8*, 1826. https://doi.org/10.1038/s41467-017-01261-5.
23. Schmitt, A.D., Hu, M., Jung, I., Xu, Z., Qiu, Y., Tan, C.L., Li, Y., Lin, S., Lin, Y., Barr, C.L., and Ren, B. (2016). A Compendium of Chromatin Contact Maps Reveals Spatially Active Regions in the Human Genome. Cell Rep. *17*, 2042–2059. https://doi.org/10.1016/j.celrep.2016.10.061.
24. Roadmap Epigenomics Consortium, Kundaje, A., Meuleman, W., Ernst, J., Bilenky, M., Yen, A., Heravi-Moussavi, A., Kheradpour, P., Zhang, Z., Wang, J., et al. (2015). Integrative analysis of 111 reference human epigenomes. Nature *518*, 317–330. https://doi.org/10.1038/nature14248.
25. Xu, S., Hu, E., Cai, Y., Xie, Z., Luo, X., Zhan, L., Tang, W., Wang, Q., Liu, B., Wang, R., et al. (2024). Using clusterProfiler to characterize multiomics data. Nat. Protoc. *19*, 3292–3320. https://doi.org/10.1038/s41596-024-01020-z.
26. (2025). R: A Language and Environment for Statistical Computing. Version 4.4.3 (R Foundation for Statistical Computing). https://www.R-project.org/.

27. Sayols, S. (2023). rrvgo: a Bioconductor package for interpreting lists of Gene Ontology terms. MicroPubl. Biol. *2023*, 000811. https://doi.org/10.17912/micropub.biology.000811.
28. Hemminki, K., Försti, A., Sundquist, K., Sundquist, J., and Li, X. (2017). Familial associations of lymphoma and myeloma with autoimmune diseases. Blood Cancer J. *7*, e515. https://doi.org/10.1038/bcj.2016.123.
29. Che, W.I., Baecklund, F., Hellgren, K., Kuja-Halkola, R., Lundberg, I.E., Westerlind, H., and Holmqvist, M. (2023). Familial Co-Aggregation of Idiopathic Inflammatory Myopathies and Cancer: A Swedish Population-Based Study. Arthritis Rheumatol. *75*, 1445–1455. https://doi.org/10.1002/art.42493.
30. Zhang, C., Zhang, Y., Zhang, Y., and Zhao, H. (2023). Benchmarking of local genetic correlation estimation methods using summary statistics from genome-wide association studies. Brief. Bioinform. *24*, bbad407. https://doi.org/10.1093/bib/bbad407.
31. Ovsepian, S.V., O'Leary, V.B., Zaborszky, L., Ntziachristos, V., and Dolly, J.O. (2018). Synaptic vesicle cycle and amyloid beta: Biting the hand that feeds. Alzheimer's Dement. *14*, 502–513. https://doi.org/10.1016/j.jalz.2018.01.011.
32. Kocyigit, M., van Hamburg, J.P., Aronica, E., van der Kooi, A.J., Tas, S.W., and Raaphorst, J. (2026). Emerging therapies in idiopathic inflammatory myopathies. J. Neuromuscul. Dis. *13*, 553–572. https://doi.org/10.1177/22143602251384098.
33. Ward, J.M., Ambatipudi, M., O'Hanlon, T.P., Smith, M.A., de Los Reyes, M., Schiffenbauer, A., Rahman, S., Zerrouki, K., Miller, F.W., Sanjuan, M.A., et al. (2023). Shared and Distinctive Transcriptomic and Proteomic Pathways in Adult and Juvenile Dermatomyositis. Arthritis Rheumatol. *75*, 2014–2026. https://doi.org/10.1002/art.42615.
34. Parkes, J.E., Rothwell, S., Day, P.J., McHugh, N.J., Betteridge, Z.E., Cooper, R.G., Ollier, W.E., Chinoy, H., Lamb, J.A.; and Myositis Genetics Consortium MYOGEN (2016). Systematic protein-protein interaction and pathway analyses in the idiopathic inflammatory myopathies. Arthritis Res. Ther. *18*, 156. https://doi.org/10.1186/s13075-016-1061-7.
35. Raouf, J., Idborg, H., Englund, P., Alexanderson, H., Dastmalchi, M., Jakobsson, P.J., Lundberg, I.E., and Korotkova, M. (2018). Targeted lipidomics analysis identified altered serum lipid profiles in patients with polymyositis and dermatomyositis. Arthritis Res. Ther. *20*, 83. https://doi.org/10.1186/s13075-018-1579-y.
36. Loell, I., Raouf, J., Chen, Y.W., Shi, R., Nennesmo, I., Alexanderson, H., Dastmalchi, M., Nagaraju, K., Korotkova, M., and Lundberg, I.E. (2016). Effects on muscle tissue remodeling and lipid metabolism in muscle tissue from adult patients with polymyositis or dermatomyositis treated with immunosuppressive agents. Arthritis Res. Ther. *18*, 136. https://doi.org/10.1186/s13075-016-1033-y.
37. Huang, W., Ren, F., Luo, L., Zhou, J., Huang, D., and Tang, L. (2021). Clinical Characteristics of Lipid Metabolism in Untreated Patients with Anti-MDA5 Antibody-Positive. Int. J. Gen. Med. *14*, 2507–2512. https://doi.org/10.2147/IJGM.S315885.
38. Wang, N., Shang, L., Liang, Z., Feng, M., Wang, Y., Gao, C., and Luo, J. (2024). Altered metabolic profiles of dermatomyositis with different myositis-specific autoantibodies associated with clinical phenotype. Front. Immunol. *15*, 1429010. https://doi.org/10.3389/fimmu.2024.1429010.
39. Huo, T., Yuan, X., Han, J., Shi, J., Xiong, Y., Tian, F., Xu, Z., Cai, M., Xu, Y., Chen, H., et al. (2023). Serum metabolomic analysis reveals disorder of steroid hormone biosynthesis in patients with idiopathic inflammatory myopathy. Front. Immunol. *14*, 1188257. https://doi.org/10.3389/fimmu.2023.1188257.
40. Plaisier, E., Gribouval, O., Alamowitch, S., Mougenot, B., Prost, C., Verpont, M.C., Marro, B., Desmettre, T., Cohen, S.Y., Roullet, E., et al. (2007). COL4A1 mutations and hereditary angiopathy, nephropathy, aneurysms, and muscle cramps. N. Engl. J. Med. *357*, 2687–2695. https://doi.org/10.1056/NEJMoa071906.
41. Trouillet, A., Lorach, H., Dubus, E., El Mathari, B., Ivkovic, I., Dégardin, J., Simonutti, M., Paques, M., Guillonneau, X., Sennlaub, F., et al. (2017). Col4a1 mutation generates vascular abnormalities correlated with neuronal damage in a mouse model of HANAC syndrome. Neurobiol. Dis. *100*, 52–61. https://doi.org/10.1016/j.nbd.2016.12.014.
42. Labelle-Dumais, C., Schuitema, V., Hayashi, G., Hoff, K., Gong, W., Dao, D.Q., Ullian, E.M., Oishi, P., Margeta, M., and Gould, D.B. (2019). COL4A1 Mutations Cause Neuromuscular Disease with Tissue-Specific Mechanistic Heterogeneity. Am. J. Hum. Genet. *104*, 847–860. https://doi.org/10.1016/j.ajhg.2019.03.007.
43. Suzuki, Y., Ménager, H., Brancotte, B., Vernet, R., Nerin, C., Boetto, C., Auvergne, A., Linhard, C., Torchet, R., Lechat, P., et al. (2024). Trait selection strategy in multi-trait GWAS: Boosting SNP discoverability. HGG Adv. *5*, 100319. https://doi.org/10.1016/j.xhgg.2024.100319.

HGGA, Volume 7

Supplemental information

Identification of genetic variants associated with idiopathic inflammatory myopathies via cross-trait analysis with B cell lymphomas

Weng Ian Che, James N. Jarvis, Anton Öberg Sysojev, Catherine Zhu, Karina Patasova, International Lymphoma Epidemiology Consortium (InterLymph), IMACS Genetics Group (MYOGEN), Karin E. Smedby, Ingrid E. Lundberg, Helga Westerlind, Janine A. Lamb, and Marie Holmqvist

Identification of genetic variants associated with idiopathic inflammatory myopathies via cross-trait analysis with B-cell lymphomas

Weng Ian Che[1], James N. Jarvis[2], Anton Öberg Sysojev[3], Catherine Zhu[4], Karina Patasova[3], International Lymphoma Epidemiology Consortium (InterLymph)#, IMACS Genetics Group (MYOGEN)#, Karin E. Smedby[3, 5], Ingrid E. Lundberg[6, 7], Helga Westerlind[3], Janine A Lamb*[8], Marie Holmqvist*[3, 7]

[1]Department of Public Health and Medicinal Administration, Faculty of Health Sciences, University of Macau, Macau SAR, China. [2]Department of Pediatrics and Center for Indigenous Health, University of Washington School of Medicine, Seattle, WA, USA. [3]Clinical epidemiology division, Department of Medicine, Solna, Karolinska Institutet, Stockholm, Sweden. [4]Institute for Clinical and Translational Research, Baylor College of Medicine, Houston, TX, USA. [5]Hematology Center, Karolinska University Hospital, Stockholm, Sweden. [6]Division of rheumatology, Department of Medicine, Solna, Karolinska Institutet, Stockholm, Sweden. [7]ME Gastro, Derm and Rheuma, Theme Inflammation and Aging, Karolinska University Hospital, Stockholm, Sweden. [8]Epidemiology and Public Health Group, School of Health Sciences, The University of Manchester, Manchester, UK.

*Dr. Janine A Lamb and Dr. Marie Holmqvist contributed equally to this work.

#The contributing members of the disease consortia are listed in the supplementary appendices.

Corresponding author

Marie Holmqvist

Address: Department of Medicine, Solna, Eugeniahemmet, T2, Karolinska Universitetssjukhuset Solna 171 76 Stockholm, Sweden

Email: marie.holmqvist@ki.se

ORCiD: https://orcid.org/0000-0001-8996-5260

Supplementary materials

SUPPLEMENTARY METHODS

Quality control and standardization

Summary statistics data of IIM and B-cell lymphoma subtypes were quality controlled and standardized. For the MYOGEN data, we filtered out SNPs that were multiallelic or located in conversion unstable regions (defined according to the published list of conversion unstable regions in GRCh38[1]). We lifted SNPs from the MYOGEN data to GRCh37 from GRCh38 using rtracklayer::liftOver in R[2,3] and SNPs that failed to convert were excluded. For the InterLymph data, we filtered duplicated SNPs and SNPs with association testing computed from a single study group. We used the R package MungeSumstats to standardise summary statistics from the MYOGEN and InterLymph consortia and computed Z scores of SNPs based on p-values.[4,5] In the standardisation, reference alleles were aligned to the 1000 Genomes phase 2 Reference Genome Sequence in GRCh37. Z scores were flipped if effective alleles rather than the reference alleles were aligned. SNPs that were ambiguous, unmatched to or missing on the reference genome, or with null effect size were filtered.

SUPPLEMENTARY TABLES

Table S1. Number of cases, controls, and single nucleotide polymorphisms (SNPs) included after in quality control and data standardization

		MYOGEN		InterLymph			
	**Number of SNPs/patients**	**Meta-analysis DM**	**Meta-analysis PM**	**DLBCL**	**FL**	**CLL**	**MZL**
**Cases**		1,131	1,094	3,587	2,847	3,100	825
**Controls**		11,697	11,697	7,666	8,107	7,667	6,221
	**Imputed SNPs**	1,255,706	1,254,738	9,116,853	9,078,855	9,098,434	8,478,065
**Stable biallelic SNPs**	Single-study group	-	-	498,560	460,723	487,417	-
	SNP duplicates	-	-	2	8	3	-
	Missing OR	-	-	-	-	-	-
	Multiallelic	83,756	83,692	-	-	-	-
**LiftOver to GRCh37**	Conversion unstable SNPs	50	50	-	-	-	-
	Failed to be converted	836	836	-	-	-	-
	Missing on reference genome	74	74	1,228	1,230	1,230	1,212
**QC and Alignment**	Ambiguous SNPs	177,921	177,751	1,330,560	1,330,713	1,329,637	1,309,967
	Zero effect value	-	-	6,773	6,252	6,398	3,776
	Not matched to reference genome	69	69	-	-	-	-
**Qced SNPs**	**After QC and alignment**	**993,000**	**992,266**	**7,279,730**	**7,279,929**	**7,273,749**	**7,163,110**

DM: Dermatomyositis; PM: Polymyositis; DLBCL: Diffuse large B-cell lymphoma; FL: Follicular lymphoma; CLL: Chronic lymphocytic leukaemia; MZL: Marginal zone lymphoma.

Table S2. Number of single nucleotide polymorphisms for each disease pair included in the conditional false discovery rate (condFDR) and multi-trait association of GWAS (MTAG) analyses

	Number of SNPs analysed	
	**CondFDR**	**MTAG**
**DM-DLBCL**	833,376	889,442
**DM-FL**	833,458	889,442
**DM-CLL**	833,318	889,442
**DM-MZL**	831,324	889,442
**PM-DLBCL**	833,281	889,198
**PM-FL**	833,377	889,198
**PM-CLL**	833,240	889,198
**PM-MZL**	831,242	889,198

GWAS: Genome-wide association study; DM: Dermatomyositis; PM: Polymyositis; DLBCL: Diffuse large B-cell lymphoma; FL: Follicular lymphoma; CLL: Chronic lymphocytic leukaemia; MZL: Marginal zone lymphoma.

Table S3. Parameter settings in FUMA SNP2GENE

**Parameters for candidate SNPs identification for all identified SNPs**	
r2	0.8
Reference panel	1KG/Phase3 EUR
Include variants in reference panel (non-GWAS tagged SNPs in linkage disequilibrium, LD)	Yes
Maximum distance between LD blocks to merge into a locus (< kb)	250
	Positional mapping
Maximum distance to genes (kb)	10
CADD, Regulome or 15-core chromatin state filtering	No
**eQTL mapping for newly identified SNPs**	
Tissue type	eQTLcatalogue/Alasoo_2018_ge_macrophage_IFNg_Salmonella.txt.gz, eQTLcatalogue/Alasoo_2018_ge_macrophage_IFNg.txt.gz, eQTLcatalogue/Alasoo_2018_ge_macrophage_naive.txt.gz, eQTLcatalogue/Alasoo_2018_ge_macrophage_Salmonella.txt.gz, eQTLcatalogue/BLUEPRINT_ge_monocyte.txt.gz, eQTLcatalogue/BLUEPRINT_ge_neutrophil.txt.gz, eQTLcatalogue/BLUEPRINT_ge_T-cell.txt.gz, eQTLcatalogue/BrainSeq_ge_brain.txt.gz, eQTLcatalogue/CEDAR_B-cell_CD19.txt.gz, eQTLcatalogue/CEDAR_ileum.txt.gz, eQTLcatalogue/CEDAR_monocyte_CD14.txt.gz, eQTLcatalogue/CEDAR_neutrophil_CD15.txt.gz, eQTLcatalogue/CEDAR_platelet.txt.gz, eQTLcatalogue/CEDAR_rectum.txt.gz, eQTLcatalogue/CEDAR_T-cell_CD4.txt.gz, eQTLcatalogue/CEDAR_T-cell_CD8.txt.gz, eQTLcatalogue/CEDAR_transverse_colon.txt.gz, eQTLcatalogue/Fairfax_2012_B-cell_CD19.txt.gz, eQTLcatalogue/Fairfax_2014_IFN24.txt.gz, eQTLcatalogue/Fairfax_2014_LPS24.txt.gz, eQTLcatalogue/Fairfax_2014_LPS2.txt.gz, eQTLcatalogue/Fairfax_2014_naive.txt.gz, eQTLcatalogue/GENCORD_ge_fibroblast.txt.gz, eQTLcatalogue/GENCORD_ge_LCL.txt.gz,

eQTLcatalogue/GENCORD_ge_T-cell.txt.gz, eQTLcatalogue/GEUVADIS_ge_LCL.txt.gz,
eQTLcatalogue/HipSci_ge_iPSC.txt.gz, eQTLcatalogue/Kasela_2017_T-cell_CD4.txt.gz,
eQTLcatalogue/Kasela_2017_T-cell_CD8.txt.gz,
eQTLcatalogue/Lepik_2017_ge_blood.txt.gz,
eQTLcatalogue/Naranbhai_2015_neutrophil_CD16.txt.gz,
eQTLcatalogue/Nedelec_2016_ge_macrophage_Listeria.txt.gz,
eQTLcatalogue/Nedelec_2016_ge_macrophage_naive.txt.gz,
eQTLcatalogue/Nedelec_2016_ge_macrophage_Salmonella.txt.gz,
eQTLcatalogue/Quach_2016_ge_monocyte_IAV.txt.gz,
eQTLcatalogue/Quach_2016_ge_monocyte_LPS.txt.gz,
eQTLcatalogue/Quach_2016_ge_monocyte_naive.txt.gz,
eQTLcatalogue/Quach_2016_ge_monocyte_Pam3CSK4.txt.gz,
eQTLcatalogue/Quach_2016_ge_monocyte_R848.txt.gz,
eQTLcatalogue/Schwartzentruber_2018_ge_sensory_neuron.txt.gz,
eQTLcatalogue/TwinsUK_ge_blood.txt.gz, eQTLcatalogue/TwinsUK_ge_fat.txt.gz,
eQTLcatalogue/TwinsUK_ge_LCL.txt.gz, eQTLcatalogue/TwinsUK_ge_skin.txt.gz,
eQTLcatalogue/van_de_Bunt_2015_ge_pancreatic_islet.txt.gz, scRNA_eQTLs/B_cell.txt.gz,
scRNA_eQTLs/DC.txt.gz, scRNA_eQTLs/NK.txt.gz, scRNA_eQTLs/Monocyte.txt.gz,
scRNA_eQTLs/Classical_Monocyte.txt.gz, scRNA_eQTLs/Non_classical_Monocyte.txt.gz,
scRNA_eQTLs/T_CD4.txt.gz, scRNA_eQTLs/T_CD8.txt.gz, scRNA_eQTLs/PBMC.txt.gz,
DICE/B_cell_naive.txt.gz, DICE/T_CD4_naive.txt.gz, DICE/T_CD4_naive_activated.txt.gz,
DICE/T_CD8_naive.txt.gz, DICE/T_CD8_naive_activated.txt.gz,
DICE/Monocyte_classical.txt.gz, DICE/Monocyte_non_classical.txt.gz, DICE/NK.txt.gz,
DICE/T_CD4_TFH.txt.gz, DICE/T_CD4_TH1.txt.gz, DICE/T_CD4_TH17.txt.gz,
DICE/T_CD4_TH1_17.txt.gz, DICE/T_CD4_TH2.txt.gz,
DICE/T_CD4_memory_TREG.txt.gz, DICE/T_CD4_naive_TREG.txt.gz,
eQTLGen/eQTLGen_cis_eQTLs.txt.gz, eQTLGen/eQTLGen_trans_eQTLs.txt.gz,
GTEx/v8/Cells_EBV-transformed_lymphocytes.txt.gz, GTEx/v8/Whole_Blood.txt.gz,
GTEx/v8/Colon_Sigmoid.txt.gz, GTEx/v8/Colon_Transverse.txt.gz,
GTEx/v8/Esophagus_Gastroesophageal_Junction.txt.gz, GTEx/v8/Esophagus_Mucosa.txt.gz,
GTEx/v8/Esophagus_Muscularis.txt.gz, GTEx/v8/Lung.txt.gz,

	GTEx/v8/Muscle_Skeletal.txt.gz, GTEx/v8/Cells_Cultured_fibroblasts.txt.gz, GTEx/v8/Skin_Not_Sun_Exposed_Suprapubic.txt.gz, GTEx/v8/Skin_Sun_Exposed_Lower_leg.txt.gz, GTEx/v8/Small_Intestine_Terminal_Ileum.txt.gz, GTEx/v8/Spleen.txt.gz
P-value threshold	FDR < 0.05
CADD, Regulome or 15-core chromatin state filtering	No
**3D Chromatin interaction mapping for newly identified SNPs**	
Builtin chromatin interaction data	EP/PsychENCODE/EP_links_oneway.txt.gz, HiC/PsychENCODE/Promoter_anchored_loops.txt.gz, HiC/GSE87112/Lung.txt.gz, HiC/GSE87112/Small_Bowel.txt.gz, HiC/GSE87112/Spleen.txt.gz, HiC/GSE87112/GM12878.txt.gz
P-value threshold	FDR < 1 x 10^{-6}
Promoter region window	250bp upstream and 500bp downstream from transcriptional start site
Annotate enhancer/promoter regions (Roadmap 111 epigenomes)	E029, E030, E031, E032, E033, E034, E035, E036, E037, E038, E039, E040, E041, E042, E043, E044, E045, E046, E047, E048, E050, E051, E062, E075, E076, E106, E077, E078, E079, E084, E085, E109, E101, E102, E103, E092, E094, E110, E111, E017, E088, E096, E052, E089, E100, E107, E108, E055, E056, E057, E058, E059, E061, E113, E093, E112
Filter SNPs by enhancers	Yes
Filter genes by promoters	Yes
CADD, Regulome or 15-core chromatin state filtering	No
**Gene types**	
Ensemble version	v102
Gene type	protein_coding
**Others**	
Major histocompatibility complex exclusion	Yes for novel SNPs in all mappings

Table S4. Loci associated with dermatomyositis and polymyositis risk identified by conditioning on four common B-cell lymphoma subtypes, at genome-wide significance reported in previous studies

Disease	Conditioning disease	SNP ID	CHR:BP in hg19	Function	Reference allele	Effect allele	Z score	P-value§	Condfdr	Nearest protein coding genes#
**DM**	DLBCL, FL, CLL, MZL	rs12203592	6:396321	intronic	C	T	6.03	1.13E-08	7.41E-07	***IRF4***
	DLBCL, FL, CLL, MZL	rs9273371	6:32626565	upstream	C	T	13.90	1.31E-39	7.26E-29	*HLA-DQB1*
**PM**	DLBCL, FL, CLL, MZL	rs2476601	1:114377568	exonic	A	G	-6.20	1.44E-08	1.43E-04	***PTPN22***, *PHTF1, RSBN1*
	DLBCL, FL, CLL, MZL	rs74925618	2:191377783	intronic	T	C	5.86	8.19E-08	4.34E-04	***NEMP2***, *HIBCH, INPP1, MFSD6*
	DLBCL, FL, CLL, MZL	rs2523495	6:31377978	intronic	C	T	17.41	4.47E-57	4.82E-29	***MICA***

SNP: Single nucleotide polymorphism; GWAS: Genome-wide association study; DLBCL: Diffuse large B-cell lymphoma; FL: Follicular lymphoma; CLL: Chronic lymphocytic leukaemia; MZL: Marginal zone lymphoma; CHR:BP: Chromosome:Base pair; condfdr: Conditional false discovery rate

§ The p-values were adjusted using intergenic inflation control.

#The lead SNPs and non-GWAS-tagged candidate SNPs in linkage disequilibrium ($r^2 \geq 0.8$) with the lead SNPs were mapped in this positional mapping. The gene containing a lead SNP is highlighted in bold.

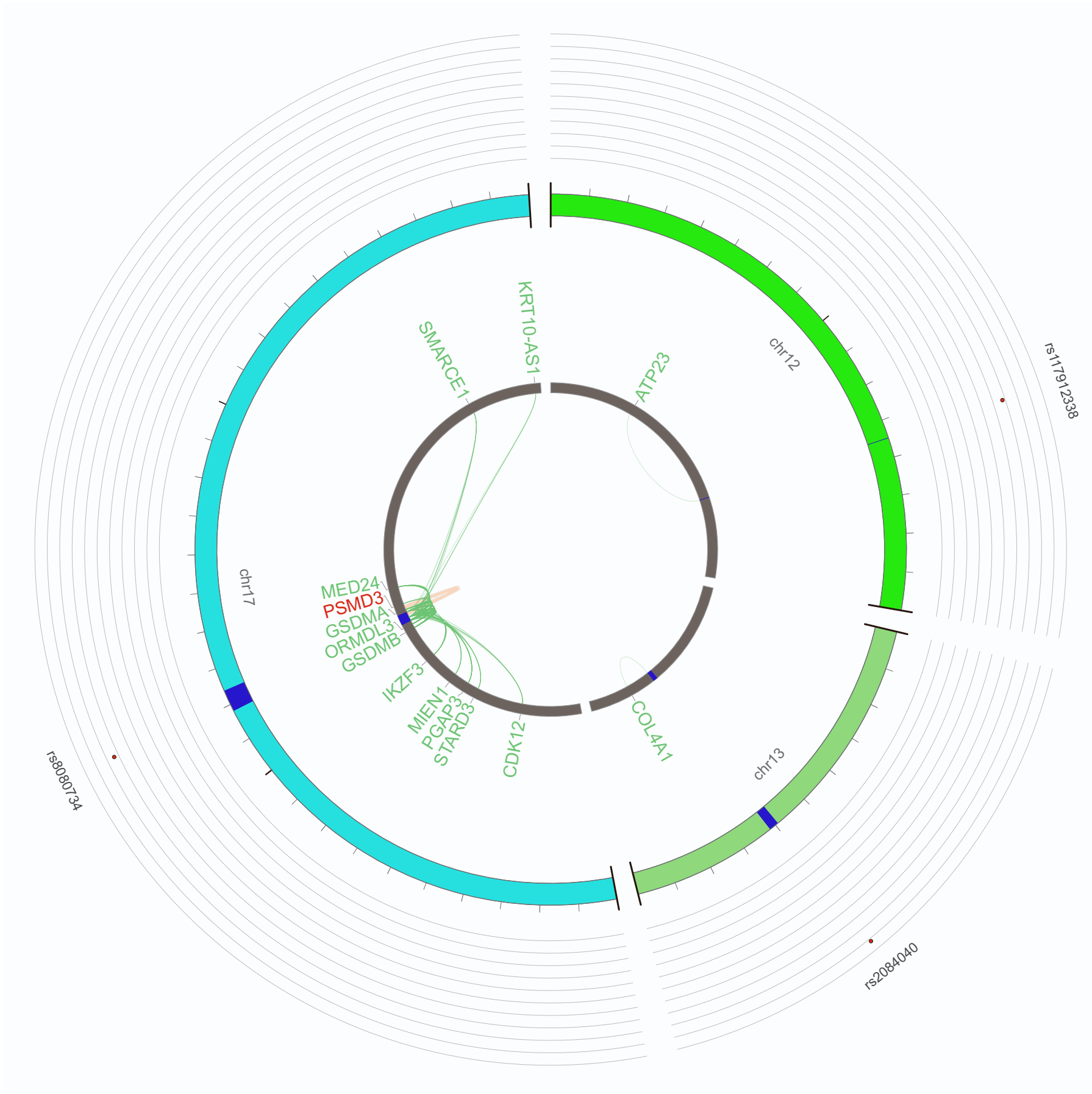

Supplementary Figure S1. eQTLs and Hi-C chromatin interactions at novel DM risk loci. Data is presented for risk loci on chromosomes 12, 13, and 17, arranged clockwise. The outermost outer layer is the Manhattan plot displaying the lead SNPs with SNP ID labelled. The outer circle is the chromosome coordinate with genomic risk loci are highlighted in blue. Genes mapped by either eQTLs (green), chromatin interaction (orange) or both (red) are shown on the inner circle. eQTL and chromatin interaction links are shown as links in green and orange, respectively. DM: Dermatomyositis; SNP: Single nucleotide polymorphism; eQTL: expression quantitative trait locus.

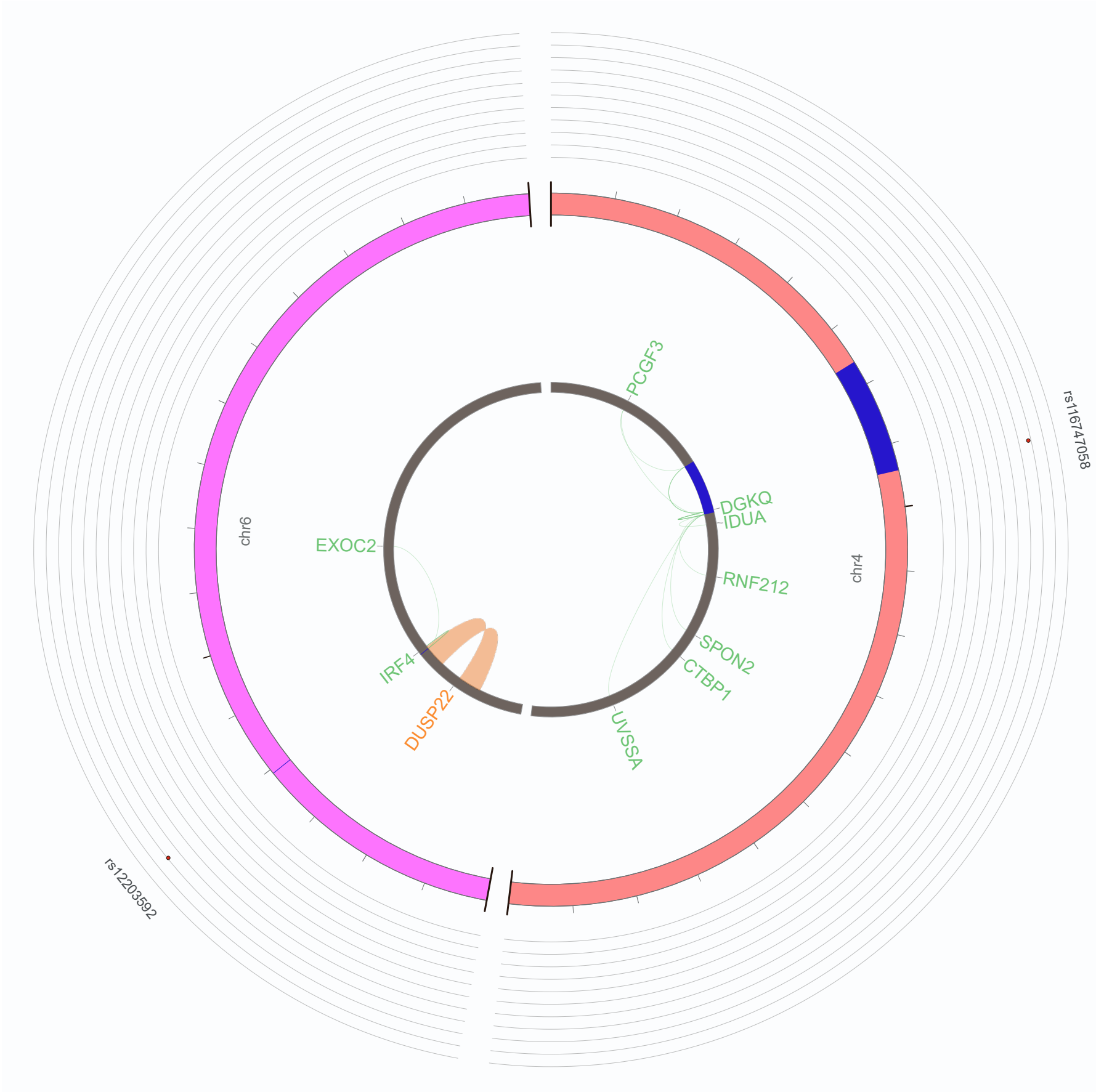

Supplementary Figure S2. eQTLs and Hi-C chromatin interactions at novel PM risk loci. Data is presented for risk loci on chromosomes 4, and 6, arranged clockwise. The outermost layer is the Manhattan plot displaying the lead SNPs with SNP ID labelled. The outer circle is the chromosome coordinate with genomic risk loci are highlighted in blue. Genes mapped by either eQTLs (green) or chromatin interaction (orange) are shown on the inner circle. eQTL and chromatin interaction links are shown as links in green and orange, respectively. PM: Polymyositis; SNP: Single nucleotide polymorphism; eQTL: expression quantitative trait locus.

SUPPLEMENTARY APPENDIX I: Investigators from IMACS Myositis Genetics Scientific Interest Group (MYOGEN) contributing to MYOGEN GWAS and ImmunoChip studies

Chris Amos. Baylor College of Medicine, Houston, Texas.

Olivier Benveniste. Department of Internal Medicine and Clinical Immunology, Pitié-Salpêtrière Hospital, Paris, France.

Hector Chinoy. National Institute for Health Research Manchester Biomedical Research Centre, Manchester University NHS Foundation Trust, The University of Manchester, Manchester, UK, and Department of Rheumatology, Salford Royal Hospital, Northern Care Alliance NHS Foundation Trust, Manchester Academic Health Science Centre, Salford, UK, and Centre for Musculoskeletal Research, Faculty of Biology, Medicine and Health, The University of Manchester, Manchester, UK.

Jan L De Bleecker, Boel De Paepe. Department of Neurology, Ghent University, Ghent, Belgium.

Andrea Doria. Rheumatology Unit, Department of Medicine, University of Padova, Padova, Italy.

Zoltan Geiger. University of Debrecen, Faculty of Medicine, Department of Internal Medicine, Division of Clinical Immunology, Debrecen, Hungary.

Peter Gregersen. The Robert S. Boas Center for Genomics and Human Genetics, The Feinstein Institute, Manhasset, New York.

Michael G. Hanna. Department of Neuromuscular Diseases, UCL Queen Square Institute of Neurology, and Centre for Rheumatology, UCL Division of Medicine, University College London, London, UK.

Janine A Lamb. Epidemiology and Public Health Group, School of Health Sciences, University of Manchester, Manchester, UK.

Vidya Limaye. Rheumatology Unit, Royal Adelaide Hospital and Discipline of Medicine, Adelaide University, Adelaide, Australia.

Ingrid Lundberg. Division of Rheumatology, Department of Medicine, Solna, Karolinska Institutet, Karolinska University Hospital, Stockholm, Sweden.

Pedro Machado. Department of Neuromuscular Diseases, UCL Queen Square Institute of Neurology, and Centre for Rheumatology, UCL Division of Medicine, University College London, London, UK.

Pernille Mathiesen. Paediatric Department, Slagelse Hospital and Paediatric Rheumatology Unit, Rigshospitalet, Copenhagen, Denmark.

Britta Maurer. Department of Rheumatology and Immunology, University Hospital, Bern, Switzerland.

Frederick W. Miller. Environmental Autoimmunity Group, National Institute of Environmental Health Sciences, NIH, Bethesda, Maryland.

Øyvind Molberg. Department of Rheumatology, Oslo University Hospital, Oslo, Norway.

Lauren Pachman. Ann & Robert H. Lurie Children's Hospital of Chicago, Northwestern University Feinberg School of Medicine, Chicago, Illinois.

Leonid Padyukov: Division of Rheumatology, Department of Medicine, Solna, Karolinska Institutet, Karolinska University Hospital, Stockholm, Sweden.

Timothy Radstake. Department of Rheumatology and Clinical Immunology, University Medical Center, Utrecht, the Netherlands.

Ann M Reed. Department of Pediatrics, Duke University, Durham, North Carolina.

Lisa G. Rider. Environmental Autoimmunity Group, National Institute of Environmental Health Sciences, NIH, Bethesda, Maryland.

Simon Rothwell: Centre for Genetics and Genomics Versus Arthritis, Centre for Musculoskeletal Research, Faculty of Biology, Medicine and Health, University of Manchester, Manchester, UK.

Adam Schiffenbauer. Environmental Autoimmunity Group, National Institute of Environmental Health Sciences, NIH, Bethesda, Maryland.

Albert Selva-O’Callaghan. Internal Medicine Department, Vall d'Hebron General Hospital, Universitat Autonoma de Barcelona, Barcelona, Spain.

Jiri Vencovksy. Institute of Rheumatology and Department of Rheumatology, First Medical Faculty, Charles University, Prague, Czech Republic.

Lucy R Wedderburn. NIHR Biomedical Research Centre at Great Ormond Street Hospital, and Arthritis Research UK Centre for Adolescent Rheumatology, UCL Great Ormond Street Institute of Child Health, University College London, London, UK.

Catherine Zhu. Baylor College of Medicine, Houston, Texas.

SUPPLEMENTARY APPENDIX II: Investigators from InterLymph Consortium contributing to the genetics studies of B-cell lymphomas

Sonja Berndt. Division of Cancer Epidemiology and Genetics, National Cancer Institute, Rockville, MD, USA.

Parveen Bhatti. School of Population and Public Health, University of British Columbia, Vancouver, BC, V6T 1Z3, Canada. Population Health Sciences, BC Cancer Research Institute, Vancouver, BC, V5Z 1L3, Canada.

Lauren Teras. Department of Population Science, American Cancer Society, Atlanta, GA, United States.

Alain Monnereau. Bordeaux Population Health Research Center, EPICENE, UMR 1219, Univ. Bordeaux, Inserm, Bordeaux, France; Registre des Hémopathies Malignes De La Gironde - Institut Bergonié, Bordeaux, France.

Roel Vermeulen. Institute for Risk Assessment Sciences, Division of Environmental Epidemiology and Julius Center for Health Sciences and Primary Care, University Medical Center Utrecht, Utrecht University, Utrecht, Netherlands.

Delphine Casabonne. Spanish Consortium for Research on Epidemiology and Public Health (CIBERESP), Madrid, Spain; Unit of Molecular and Genetic Epidemiology in Infections and Cancer (UNIC-Molecular), Cancer Epidemiology Research Programme, IDIBELL, Catalan Institute of Oncology, Becelona, Spain.

Alexandra Nieters. Institute for Immunodeficiency, Medical Centre and Faculty of Medicine, Albert-Ludwigs-University, Freiburg, Germany.

James McKay. International Agency for Research on Cancer, World Health Organization, Lyon, France.

Brenda Birmann. Channing Division of Network Medicine, Department of Medicine, Brigham and Women's Hospital and Harvard Medical School, Boston, MA, USA.

Pierluigi Cocco. Department of Medical Sciences and Public Health, University of Cagliari, Cagliari, Italy.

James Cerhan. Department of Quantitative Health Sciences, Mayo Clinic, Rochester, MN, USA.

Brian Link. Division of Hematology, Oncology and Bone Marrow Transplantation, University of Iowa, Iowa City, IA, USA.

Roger Milne. Cancer Council Victoria, East Melbourne, Victoria, Australia; Centre for Epidemiology and Biostatistics, Melbourne School of Population and Global Health, The University of Melbourne, Victoria, Australia; Precision Medicine, School of Clinical Sciences at Monash Health, Monash University, Clayton, Victoria, Australia.

Harindra Jayasekara. Cancer Epidemiology Division, Cancer Council Victoria, Melbourne, Victoria, Australia.Centre for Epidemiology and Biostatistics, Melbourne School of Population and Global Health, The University of Melbourne, Melbourne, Victoria, Australia. School of Public Health and Preventive Medicine, Faculty of Medicine, Nursing and Health Sciences, Monash University, Clayton, Victoria, Australia.

Vijai Joseph. Memorial Sloan-Kettering Cancer Center, New York, NY, USA.

Kenneth Offit. Memorial Sloan-Kettering Cancer Center, New York, NY, USA.

Nathaniel Rothman. Division of Cancer Epidemiology and Genetics, National Cancer Institute, NIH, DHHS, Rockville, MD, USA.

Claire Vajdic. Kirby Institute, UNSW Sydney, Sydney, Australia.

Alan Arslan. Departments of Obstetrics and Gynecology and Population Health, New York University Grossman School of Medicine, New York, NY, USA.

Mark Purdue. Division of Cancer Epidemiology and Genetics, National Cancer Institute, NIH, DHHS, Rockville, MD, USA.

Henrik Hjalgrim. Haematology, Danish Cancer Institute, Copenhagen, Denmark. Department of Haematology, Rigshospitalet, University of Copenhagen, Copenhagen, Denmark. Department of Clinical Medicine, University of Copenhagen, Copenhagen, Denmark.

Karin Ekstrom Smedby. Clinical epidemiology division, Department of Medicine, Solna, Karolinska Institutet, Stockholm, Sweden. Hematology Center, Karolinska University Hospital, Stockholm, Sweden.

Paige Bracci. Department of Epidemiology and Biostatistics, University of California, San Francisco, San Francisco, California, USA.

Eleanor Kane. Epidemiology and Cancer Statistics Group, Department of Health Sciences, University of York, York, UK.

Lesley Tinker. Division of Public Health Sciences, Women's Health Initiative Clinical Coordinating Center, Fred Hutchinson Cancer Center, Seattle, WA, USA.

Qing Lan. Division of Cancer Epidemiology and Genetics, National Cancer Institute, NIH, DHHS, Rockville, MD, USA.

Susan Slager. Department of Quantitative Health Sciences, Mayo Clinic, Rochester, Minnesota, USA. Division of Hematology, Mayo Clinic, Rochester, Minnesota, USA.

Immaculata De Vivo. Channing Division of Network Medicine, Department of Medicine, Brigham and Women's Hospital and Harvard Medical School, Boston, MA, USA; Department of Epidemiology, Harvard T.H. Chan School of Public Health, Boston, MA, USA.

Gilles Salles. Department of Medicine, Lymphoma Service, Memorial Sloan Kettering Cancer Center, New York, NY, USA. Department of Medicine, Weill Cornell Medical College, New York, NY, USA.

Nicola Camp. Huntsman Cancer Institute, Salt Lake City, UT 84108, USA; Department of Internal Medicine, University of Utah School of Medicine, Salt Lake City, UT 84108, USA.

SUPPLEMENTARY APPENDIX III: Funding information for contributing GWASs

The previous GWAS and ImmunoChip studies of IIM are supported by the Association Francaise Contre Les Myopathies (AFM); European Union Sixth Framework Programme (project AutoCure; LSH-018661); European Science Foundation (ESF) in the framework of the Research Networking Programme European Myositis Network (EUMYONET); European Reference Network for Rare Neuromuscular Diseases (ERN EURO-NMD); The Swedish Research Council and grants provided by the Stockholm County Council (ALF project), the Swedish Rheumatism Association, King Gustaf V 80 year Foundation; the intramural research program of the National Institute of Environmental Health Sciences (NIEHS), the National Institutes of Health (NIH) (Project ZIA ES101074) ; Myositis UK; Arthritis Research UK (awards 18474, 21593, 21552 and 20380); Medical Research Council (MR/N003322/1); The Cure JM Foundation; the Wellcome Trust (awards 085860 and 105610); the Henry Smith Charity UK; Action Medical UK; the Remission Charity; BMA Dorris Hillier Grant 2012 and the Bath Institute of Rheumatic Diseases; Prinses Beatrix Spierfonds (W.OR12-15); the Research Fund of Region Zeeland, Denmark; the Ministry of Health, Czech Republic (Project for Conceptual Development of Research Organization grant 00023728); National Institute for Health Research (NIHR) Biomedical Research Centres (BRC) at Great Ormond Street Hospital (GOSH), Manchester and University College London Hospitals. The contributions of the NIH author(s) were made as part of their official duties as NIH federal employees, are in compliance with agency policy requirements, and are considered Works of the United States Government. However, the findings and conclusions presented in this paper are those of the author(s) and do not necessarily reflect the views of the NIH or the U.S. Department of Health and Human Services. The InterLymph consortium is

supported by the Intramural Research Program of the Division of Cancer Epidemiology and Genetics, National Cancer Institute, National Institute of Health. These funding sources were not involved in any parts of the presented research.

REFERENCES

1. Ormond C, Ryan NM, Corvin A, Heron EA. Converting single nucleotide variants between genome builds: from cautionary tale to solution. *Brief Bioinform*. Sep 2 2021;22(5)doi:10.1093/bib/bbab069
2. *liftOver: Changing genomic coordinate systems with rtracklayer::liftOver*. R package version 1.24.0; 2023. https://www.bioconductor.org/help/workflows/liftOver/
3. *R: A language and environment for statistical computing*. R Foundation for Statistical Computing; 2021. URL https://www.R-project.org/
4. Murphy AE, Schilder BM, Skene NG. MungeSumstats: a Bioconductor package for the standardization and quality control of many GWAS summary statistics. *Bioinformatics*. Dec 7 2021;37(23):4593-4596. doi:10.1093/bioinformatics/btab665
5. *MungeSumstats: Standardise summary statistics from GWAS*. R package version 1.2.4; 2022. https://github.com/neurogenomics/MungeSumstats
